# Canonical Wnt signaling in the regulation of prostate cancer stem cells: a key driver of castration-resistant prostate cancer and a therapeutic opportunity

**DOI:** 10.1186/s12967-026-08751-5

**Published:** 2026-07-31

**Authors:** Haoze Li, Hongtao Xu, Yifan Hou, Qianhui Ni, Jianhua Zhang, Yujie Ma, Xiaobo Nie, Junqing Hou

**Affiliations:** 1 Key Laboratory of Receptors-Mediated Gene Regulation, School of Basic Medical Sciences, Henan University, Kaifeng, 475004 China; 2 Henan Provincial Engineering Research Center for Precision Diagnosis and Treatment of Prostatic Diseases with Integrative Chinese-Western Medicine, Kaifeng Engineering Research Center for Minimally Invasive Diagnosis and Treatment of Prostatic Hyperplasia, Kaifeng 155 Hospital, China RongTong Medical Healthcare Group Co. Ltd., Kaifeng, 475003 China

**Keywords:** Canonical Wnt signaling pathway, Prostate cancer stem cells, Castration-resistant prostate cancer, Diagnostic markers, Epigenetic regulation, Targeted therapy

## Abstract

Prostate cancer (PCa) is the second leading cause of cancer-related death among men worldwide. Although androgen deprivation therapy (ADT) effectively suppresses tumor growth, most patients eventually progress to castration-resistant prostate cancer (CRPC) and develop metastatic disease. The self-renewal, lineage plasticity, epithelial–mesenchymal transition (EMT), and bone metastatic capacity of prostate cancer stem cells (PCSCs) are central drivers of CRPC progression, therapeutic resistance, and metastasis, and the aberrant activation of the canonical Wnt signaling pathway plays a key regulatory role in this process. This review systematically discusses PCSC markers and their applications in clinical diagnosis, elucidates the molecular mechanisms by which aberrant activation of the canonical Wnt signaling pathway, induced by the synergistic effects of genetic mutations, non-coding RNA (ncRNA) dysregulation, epigenetic dysregulation, tumor microenvironment (TME) alterations, and synergistic crosstalk with androgen receptor (AR) signaling, PI3K/AKT/mTOR signaling, Hippo–YAP/TAZ signaling, and metabolic reprogramming, enhances the stemness of PCSCs and promotes their EMT and bone metastasis. We also summarize current research on therapeutic agents targeting the canonical Wnt signaling pathway to suppress PCSCs, along with major challenges in clinical translation and potential strategies to overcome them. Collectively, we propose that targeting this pathway to inhibit PCSCs represents a highly promising strategy for controlling CRPC progression, overcoming therapeutic resistance, and ultimately improving patient outcomes.

## Graphical Abstract

**Figure:**
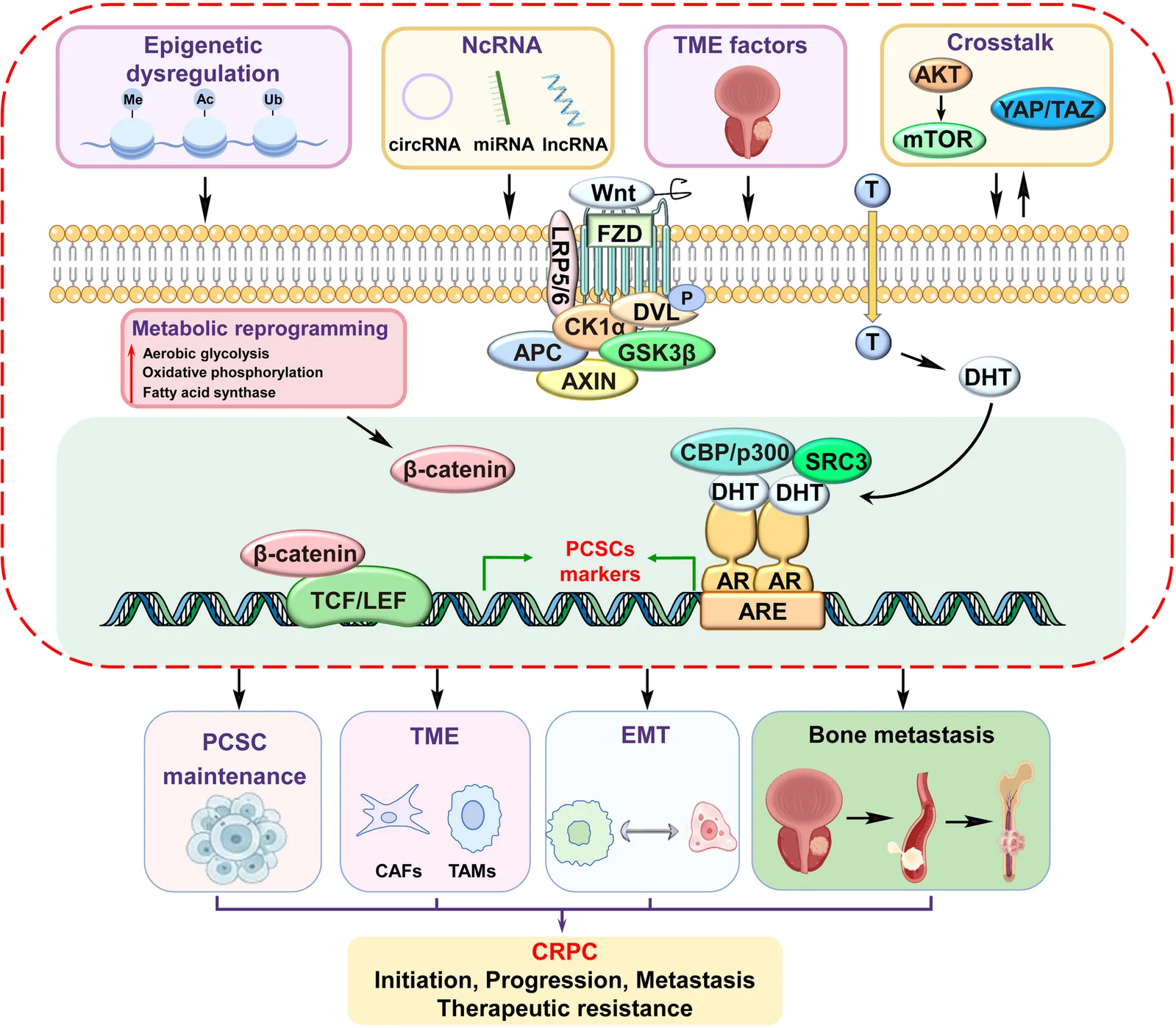

## Introduction

Prostate cancer (PCa) is the fourth most common cancer among men worldwide and the second leading cause of cancer-related mortality in men. In 2022, the number of new cases worldwide was estimated to exceed 1.46 million, with 397,000 deaths. By 2040, driven by factors such as global population aging and the widespread adoption of Western-style diets, the disease burden of PCa is projected to increase further, with the number of cases rising to approximately 2.4 million and deaths reaching 712,000, posing a grave threat to men’s health and survival [1, 2]. Based on disease progression, PCa is primarily classified into localized PCa, locally advanced PCa, and advanced PCa. Among these, advanced PCa is further subdivided into biochemically recurrent PCa, metastatic hormone-sensitive PCa, and castration-resistant PCa (CRPC) [3, 4]. Because the growth of PCa cells is highly dependent on the androgen receptor (AR) signaling pathway, androgen deprivation therapy (ADT) is currently the primary treatment strategy for PCa, effectively inhibiting tumor growth and delaying disease progression in the early stages. However, the vast majority of patients inevitably progress to CRPC following ADT [5]. Current standard of care for CRPC combines second-generation AR pathway inhibitors with continued ADT. At this stage, approximately 60% of patients progress to metastatic CRPC (mCRPC) within 3 years. Treatment options for mCRPC remain extremely limited, with chemotherapy and radiation therapy serving as the mainstays of systemic management. According to the latest data from the Mayo Clinic, the 5-year survival rate for mCRPC patients is approximately 38%, with a median overall survival (OS) of only 3 years [6]. Research indicates that the limited efficacy of current therapies stems primarily from the development of acquired therapeutic resistance in PCa cells. ADT resistance in PCa arises not from a single mechanism, but rather from multiple interrelated factors, including tumor heterogeneity, persistent AR signaling activation, and the selective expansion of pre-existing therapy-resistant cell clones [7]. Therefore, systematically elucidating the key molecular mechanisms driving the progression of PCa to CRPC is critical for identifying novel therapeutic targets, developing highly effective targeted therapies, and improving clinical outcomes.

Accumulating evidence indicates that prostate cancer stem cells (PCSCs) play a key role in the development of acquired therapeutic resistance. Their core biological properties include robust self-renewal capacity and pronounced lineage plasticity, enabling them to sustain tumor growth under therapeutic pressure and drive disease progression [8–10]. Notably, PCSCs exhibit marked cellular heterogeneity in origin: they may arise from aberrant proliferation of prostate basal cells, or alternatively, differentiated luminal epithelial cells can undergo dedifferentiation to reacquire a stem-like phenotype, thereby fueling cancer progression [11]. Furthermore, the tumor microenvironment (TME) plays a crucial regulatory role in PCSC formation. Immune cells and tumor-associated stromal cells within this microenvironment secrete cytokines and signaling molecules that induce tumor cells to acquire stem-like properties, thereby promoting PCSC generation and maintenance [12]. Dysregulation of multiple signaling pathways, including PI3K/AKT, JAK/STAT, transforming growth factor β (TGF-β), AR, Hedgehog, and Notch, contributes to the generation, maintenance, and regulation of PCSCs. Through synergistic crosstalk, these pathways collectively form the molecular basis for sustaining PCSC stemness and driving therapeutic resistance. Among these, canonical Wnt signaling is a key regulator of PCSCs, playing a central role not only in PCa but also in cancer stem cell (CSC) biology across diverse tumor types [13]. Canonical Wnt signaling drives key pathological processes, including epithelial–mesenchymal transition (EMT), bone metastasis, and TME remodeling, thereby enhancing PCSC self-renewal, proliferation, migration, and therapeutic resistance [14]. This review focuses on the regulation of canonical Wnt signaling in PCSCs and summarizes current therapeutic potential agents targeting this pathway, including their preclinical and clinical development status, to provide novel insights and potential intervention strategies for precision therapy in PCa.

### Signal transduction mechanisms of Wnt pathways

**Fig. 1:**
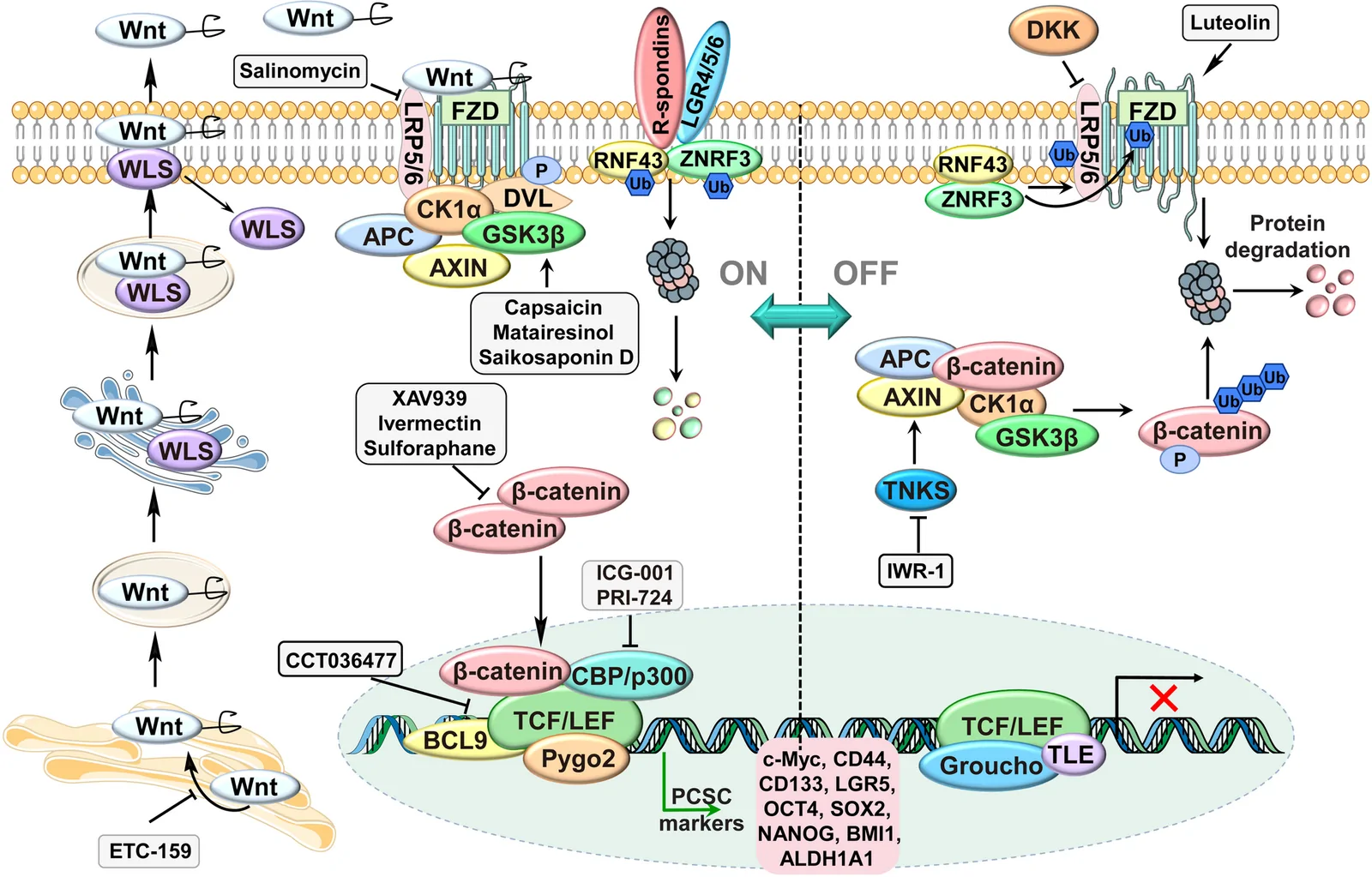
Schematic overview of the canonical Wnt signaling pathway and targeted inhibitors for PCSC inhibition. Wnt ligands undergo palmitoylation within the endoplasmic reticulum and are subsequently shuttled by the cargo receptor WLS for secretion. In the absence of Wnt stimulation, cytosolic β-catenin is phosphorylated by the destruction complex (CK1α and GSK3β), which marks it for ubiquitination and proteasomal degradation. Conversely, Wnt ligand engagement with FZD receptors and LRP5/6 co-receptors triggers the recruitment and phosphorylation of DVL. This event induces the disassembly of the destruction complex, thereby abrogating β-catenin phosphorylation and stabilizing the protein. Accumulated β-catenin then translocates to the nucleus, where it displaces the transcriptional repressors Groucho/TLE from TCF/LEF-binding elements and recruits co-activators, including BCL9, Pygo2, and CBP/p300, to orchestrate the transcription of stemness-associated target genes. RSPOs bind to LGR4/5/6 and the E3 ubiquitin ligases RNF43/ZNRF3, thereby abrogating RNF43/ZNRF3-mediated polyubiquitination and endocytic turnover of FZDs, consequently potentiating canonical Wnt signaling. Conversely, DKK proteins inhibit Wnt signaling by binding to LRP5/6, which blocks receptor complex assembly and promotes LRP5/6 internalization and lysosomal degradation. Notably, multiple therapeutic agents targeting distinct nodes of this axis for PCSC inhibition are also illustrated

The Wnt signaling pathway is a highly conserved cascade that plays a central role in embryonic development, cell proliferation, differentiation, and tissue homeostasis. Dysregulation of this pathway is closely associated with the initiation, progression, and therapeutic resistance of multiple cancers, including PCa. To date, 19 human Wnt ligands have been identified. Based on whether β-catenin serves as a central effector molecule, the Wnt signaling pathway is classified into the β-catenin-dependent canonical pathway and the β-catenin-independent non-canonical pathways. The activation of the canonical Wnt signaling pathway depends on the stability of β-catenin (Fig. 1). In the absence of Wnt ligands, the destruction complex composed of adenomatous polyposis coli (APC), AXIN, casein kinase 1α (CK1α), and glycogen synthase kinase 3β (GSK3β) phosphorylates β-catenin, leading to its ubiquitin-mediated proteasomal degradation through β-transducin repeat-containing protein (β-TrCP). Upon Wnt activation, frizzled (FZD) receptors and its co-receptors low-density lipoprotein receptor-related protein 5/6 (LRP5/6) recruit dishevelled (DVL) to destabilize the destruction complex, enabling β-catenin accumulation and nuclear translocation, where it binds to T-cell factor (TCF)/lymphoid enhancer factor (LEF) transcription factors to initiate transcription of multiple target genes [15]. In addition to Wnt ligands, the canonical Wnt pathway is regulated by several secreted modulators, among which R-spondins (RSPOs) and the Dickkopf (DKK) proteins are the two best-characterized regulators. RSPOs comprise four paralogs (RSPO1–RSPO4) and play critical roles in PCa development [16]. RSPOs bind to leucine-rich repeat–containing G protein-coupled receptors 4/5/6 (LGR4/5/6) and the E3 ubiquitin ligases ring finger protein 43 (RNF43)/zinc and ring finger 3 (ZNRF3). This dual interaction inhibits RNF43/ZNRF3-mediated polyubiquitination and endocytic turnover of FZDs, thereby potentiating Wnt signaling activity. In contrast, DKK proteins act as potent inhibitors of the canonical Wnt signaling pathway by binding LRP5/6 with high affinity to sterically block Wnt ligand–receptor complex assembly or by forming ternary complexes with the transmembrane receptor Kremen to promote internalization and lysosomal degradation of LRP5/6 [17].

Non-canonical Wnt signaling pathways, typically activated by ligands such as Wnt5a and Wnt11, are subdivided into the Wnt/planar cell polarity (PCP) pathway and the Wnt/Ca²⁺ pathway. The Wnt/PCP pathway governs cell polarity and cytoskeletal reorganization and plays essential roles in embryonic development, organogenesis, and tumor invasion [18]. The Wnt/Ca²⁺ pathway regulates intracellular Ca²⁺ flux and contributes to dorsoventral axis formation, neural tube closure, and cardiac development during embryogenesis; in adults, it modulates inflammatory responses, synaptic plasticity, and cell migration. Its dysregulation is implicated in tumor metastasis and neurodegenerative disorders. Canonical and non-canonical Wnt pathways are not mutually exclusive; they engage in extensive crosstalk at multiple regulatory nodes. For instance, binding of the non-canonical ligand Wnt5a to ROR2 induces tyrosine phosphorylation of β-catenin, thereby suppressing its nuclear translocation and transcriptional activity [19]. Moreover, Wnt5a antagonizes Wnt3a-induced canonical signaling and inhibits hematopoietic stem cell self-renewal [20]. In prostate tissue, the mutation frequency of canonical Wnt pathway components significantly exceeds that of non-canonical components. The aberrant activation of this pathway directly drives the transcription of stemness-associated genes and is therefore regarded as a core signaling axis sustaining PCSC self-renewal and stemness. Conversely, although activation of non-canonical Wnt pathways mediated by Wnt5a and Wnt11 augments invasiveness, metastatic capacity, therapeutic resistance, and transdifferentiation potential of PCa cells, it suppresses the proliferation of normal prostate epithelial stem or progenitor cells [21, 22]. Thus, selective targeting of canonical Wnt signaling represents a rational primary therapeutic strategy to disrupt the fundamental driver of PCSC stemness maintenance. Nevertheless, given the critical contribution of non-canonical Wnt signaling to PCa metastasis, future efforts should focus on combinatorial inhibition approaches and the development of dual-pathway inhibitors.

### The role of PCSCs in prostatic carcinogenesis

CSCs represent a small subpopulation within tumors that possess self-renewal capacity and multilineage differentiation potential. They drive tumor initiation, growth, and heterogeneity, and are widely regarded as the cellular origin of cancer recurrence and metastasis. CSCs were first identified in acute myeloid leukemia and later confirmed in PCa [23, 24]. The origins of CSCs are complex and multifactorial. In normal tissues, quiescent stem or progenitor cells are activated by specific growth signals; upon exposure to environmental stressors or genetic mutations, these cells may acquire aberrant proliferative and self-renewal capabilities, thereby undergoing malignant transformation into CSCs [25]. Proposed mechanisms contributing to CSC emergence include aberrant niche signaling, mutagenesis induced by chemical carcinogens, viral infection–associated genomic alterations, epigenetic dysregulation, and accumulation of endogenous DNA mutations. Moreover, chronic inflammation critically contributes to CSC formation. Under inflammatory conditions, reactive oxygen species (ROS) and reactive nitrogen species (RNS) induce oxidative and nitrosative DNA damage, generating lesions such as 8-oxo-7,8-dihydro-2′-deoxyguanosine (8-oxodG) and 8-nitroguanine [26]. Concurrently, ROS, RNS, lipid peroxidation products, and proinflammatory cytokines accumulate in the stem cell niche. Prolonged exposure of stem cells to this chronically stressed microenvironment increases their susceptibility to irreversible genomic alterations, ultimately promoting malignant transformation and CSC generation [27].

The maintenance and function of CSCs critically dependent on their specific niche, which comprises the extracellular matrix (ECM), tumor-associated macrophages (TAMs), cancer-associated fibroblasts (CAFs), and mesenchymal stem cells (MSCs). These niche components actively remodel the ECM through secretion of cytokines, chemokines, growth factors, and signaling molecules, collectively regulating CSC activity, proliferation, and stemness maintenance [28, 29]. CSCs exhibit intrinsic resistance to radiotherapy, chemotherapy, and targeted therapy, a phenomenon partly attributed to their quiescent state, enhanced DNA damage repair capacity, and upregulated drug efflux transporters. Moreover, their ability to evade therapeutic elimination renders CSCs a primary source of cancer recurrence and metastasis. Therefore, elucidating the cellular and molecular interactions within the CSC niche and identifying therapeutic strategies that selectively impair CSC self-renewal, deplete CSC populations, or induce terminal differentiation are essential for suppressing tumor progression, overcoming therapy resistance, and inhibiting invasion.

During PCa initiation and progression, PCSCs drive therapeutic resistance and CRPC development through lineage plasticity. At the core of this process, ADT imposes selective pressure that triggers molecular reprogramming in PCSCs. By reactivating embryonic transcription factors such as octamer-binding transcription factor 4 (OCT4, encoded by *POU5F1* gene), SRY-box transcription factor 2 (SOX2), NANOG, they undergo cellular reprogramming to acquire stem-like properties, dedifferentiate, and transdifferentiate toward neuroendocrine lineages. Concurrently, alternative signaling pathways such as YAP/TAZ, Wnt, Notch, Hedgehog , TGF-β, and STAT3 are activated, conferring survival and proliferative advantages to AR-negative PCSC subpopulations. This plasticity drives tumor evolution toward androgen-independent states, including amphicrine intermediate cells co-expressing AR and neuroendocrine markers [30], highly invasive AR-negative neuroendocrine prostate cancer (NEPC) driven by ASCL1 [31], and AR-negative mesenchymal and stem-like PCa subtypes [32]. During lineage conversion, PCSCs also acquire intrinsic resistance traits. For instance, the YAP/Nicotinamide phosphoribosyltransferase (NAMPT) axis promotes a highly invasive, stem cell–like CRPC subtype sensitive to NAMPT inhibitors [33]. Similarly, Ca²⁺/calmodulin-dependent protein kinase 1D (CAMK1D) activates the AMPK/PINK1 pathway to stimulate mitophagy, thereby facilitating PCSC expansion and enzalutamide resistance [34]. Moreover, a subset of PCSCs enters a quiescent, low-proliferation state, evading immune surveillance and cytotoxic therapies, and serves as reservoirs for tumor recurrence. Thus, therapeutic resistance in PCa and CRPC development represent evolutionary adaptations driven by PCSC lineage plasticity and synergized by the TME. Consequently, to eradicate PCSCs, overcome therapeutic resistance, and control CRPC, it is essential to concurrently inhibit AR signaling and combine this with interventions that either block key plasticity-driving pathways or induce terminal differentiation.

### PCSC markers of and their application in PCa diagnosis

#### Markers of PCSCs

In recent years, numerous studies have identified diverse PCSC markers across multiple categories, including cell surface proteins, transcription factors, metabolic enzymes, and ATP binding cassette (ABC) transporters (Table 1). CD44 is widely recognized as a core PCSC marker. ADT induces its upregulation; CD44⁺ subpopulations exhibit enhanced tumor-initiating and self-renewal capacity and promote CRPC development, indicating a critical role for CD44 in CRPC pathogenesis [35]. CD133 (encoded by *PROM1*), another established stem cell marker, is commonly used in combination with CD44 to enrich tumor-initiating cell populations [36, 37]. α2β1 integrin, a basal epithelial cell marker, contributes to maintenance of the PCSC niche. In PCa, elevated β1 integrin expression is positively correlated with enhanced anti-apoptotic activity and metastatic potential of tumor cells [38]. Moreover, α2β1 integrin(high)/CD133⁺ PCSCs exhibit increased radioresistance [39]. Although co-expression of α2β1 integrin with CD133 further improves enrichment efficiency, the presence of α2β1 integrin in stromal cells limits its specificity for PCSC identification [40]. Trop-2 is highly expressed in PCa tissue, and Trop-2⁺/CD133⁺ cells are detected in both benign and malignant regions, supporting identification of the PCSC niche. Nevertheless, stromal Trop-2 expression compromises its utility as a PCSC-specific marker [40]. Additionally, CD24 enhances stemness properties and drives bone metastasis via canonical Wnt pathway activation and is considered a key driver of PCa progression and metastasis as well as a promising anti-metastatic therapeutic target [41]. EpCAM is upregulated in KRAS-mutant–induced PCSCs and serves as a surface marker distinguishing tumor-initiating cells from differentiated progeny [41]. Aldehyde dehydrogenase 1 family member A1 (ALDH1A1) is a key functional marker of PCSCs. Cell subpopulations with high ALDH1A1 expression exhibit enhanced tumor-initiating capacity, self-renewal activity, and chemotherapy resistance. In enzalutamide-resistant PCa cells, ALDH1A1 expression is significantly elevated and can be used in combination with CD44 to distinguish distinct androgen-independent PCSC subpopulations [42]. Immunohistochemical analysis of clinical tissue specimens reveals that ALDH1A1 expression is markedly low and confined to basal epithelial cells in normal prostate tissue, but is robustly upregulated in secretory-type adenocarcinoma and neuroendocrine carcinoma cells. High ALDH1A1 expression is significantly associated with higher Gleason scores, advanced pathological stage, and poorer OS and cancer-specific survival, underscoring its potential utility as a prognostic biomarker [43].

**Table 1:** Main markers of PCSCs and their function

Marker	Function	References
CD44	Confers enhanced tumor-initiating and self-renewal capacities, and promote CRPC development.	[35]
CD133 (PROM1)	Classic stem cell surface markers; co-utilizes with CD44 to enrich PCSC populations.	[36]
α2β1 integrin	Prostates basal cell-associated markers, maintains the PCSC niche; co-utilizes with CD133 to improve PCSC enrichment efficiency.	[40]
Trop-2	Expresses in prostate basal cells, present in both benign and malignant regions.	[40]
CD24	Potentiates CSC properties and drives bone metastasis through activating the canonical Wnt signaling.	[41]
EpCAM	An epithelial cell adhesion molecule that distinguishes tumor-initiating cells from differentiated counterparts.	[41]
ALDH1A1	Enhances tumor-initiating capacity and self-renewal activity, while mediates chemoresistance.	[42]
OCT4 (POU5F1)	Maintains the undifferentiated state and self-renewal capacity of cells.	[42]
NANOG		
SOX2	Maintains the undifferentiated state and self-renewal capacity of cells. Drives the transdifferentiation of prostate adenocarcinoma toward neuroendocrine phenotype during TP53 and RB1 inactivation.	[42, 44]
c-Myc	Governs cell proliferation, metabolic reprogramming, and stemness maintenance.	[46]
BMI1	A polycomb group member that sustains stem cell self-renewal and suppresses senescence.	[42]
ABCG2	Effluxes chemotherapeutic agents, protects stem cells from drug-induced damage and preserves the undifferentiated state of PCSCs.	[47, 48]
ABCB1	Enhances drug resistance in stem cells.	[50]

Reactivation of embryonic stem cell pluripotency-associated transcription factors in PCSCs represents a key molecular event driving lineage plasticity and therapeutic resistance. OCT4, SOX2, and NANOG act synergistically in PCSCs to maintain their undifferentiated state and self-renewal capacity. In enzalutamide-resistant cell lines and PCa specimens after ADT, OCT4, SOX2, and NANOG expression is significantly upregulated, indicating strong association with therapeutic resistance [42]. Among these, SOX2 has been established as a central driver of lineage plasticity. Upon TP53 and RB1 inactivation, SOX2 overexpression confers enzalutamide resistance and drives transdifferentiation of prostate adenocarcinoma cells to neuroendocrine carcinoma [44]. Elevated NANOG mRNA levels in PCa tissues are positively correlated with preoperative prostate specific antigen (PSA) concentration, tumor volume, and regional lymph node metastasis [45]. Although OCT4 and Krüppel-like factor 4 (KLF4) can also be induced, neither alone nor in combination confers a drug-resistant phenotype, underscoring SOX2’s non-redundant role in PCSC lineage reprogramming. *C-MYC*, a canonical oncogene, is highly expressed in PCSCs and drives proliferation, metabolic reprogramming, and stemness maintenance [46]; however, its widespread expression across proliferating cell types limits its specificity as a standalone PCSC marker. Furthermore, BMI1, a Polycomb group protein, maintains stem cell self-renewal and suppresses senescence, and is significantly upregulated in drug-resistant PCa cell lines [42].

Overexpression of ABC transporters underpins the intrinsic chemoresistance of PCSCs, enabling active efflux of cytotoxic drugs and thereby shielding these cells from therapeutic damage. ATP binding cassette subfamily G member 2 (ABCG2) is a central mediator of this mechanism; its efflux of protoporphyrin IX significantly reduces the sensitivity of PCa cells to 5-aminolevulinic acid–based photodynamic therapy [47]. In CRPC specimens and ADT–resistant PCa tissues, ABCG2 expression is markedly upregulated, and by promoting androgen efflux, it helps sustain the undifferentiated, stem-like state of PCSCs. Pharmacological inhibition of ABCG2 restores PCSC sensitivity to ADT, supporting its potential as a therapeutic target for reversing PCSC-mediated resistance [48]. The expression of stem cell markers (e.g., ABCG2, SOX2, OCT4) is significantly upregulated in recurrent PCa tissues, and its quantitative assessment can predict chemotherapy sensitivity as well as postoperative recurrence risk [49]. Multidrug resistance protein 1 (MDR1) is also overexpressed in PCSCs and correlates with Notch pathway activation. Notch inhibition reduces MDR1 expression and restores docetaxel sensitivity in resistant PCa cells [50]. Collectively, these markers not only sustain PCSC self-renewal and multipotent differentiation but also critically drive lineage plasticity, therapeutic resistance, and metastasis. Moreover, they represent promising targets for PCa diagnosis, prognosis assessment, and targeted intervention.

#### The application of PCSC markers in PCa diagnosis

Currently, these markers have demonstrated potential clinical utility for PCSC identification, PCa diagnosis, and prognostic stratification, and are therefore emerging as promising biomarkers for precision diagnosis and prognosis of PCa. For example, CD44 expression in prostate tumor tissue correlates positively with Gleason score and contributes to clinical risk stratification [51]. However, the broad expression of CD44 in both benign prostatic epithelium and malignant cells limits its specificity as a standalone marker, given that CSCs are defined as a minor subpopulation (< 10%) of total tumor cells. Consequently, CD44 detection alone is insufficient for reliable clinical discrimination. In contrast, combined detection of the embryonic stem cell markers EZH2 and SOX2 markedly improves PCSC identification efficiency [52]. Moreover, PCSC marker profiles can predict differential responses to radiotherapy and chemotherapy: elevated CD44 expression confers radioresistance, whereas CD44 knockdown enhances radiosensitivity by impairing double-strand DNA break repair [53]. Critically, following radiotherapy or chemotherapy, the proportion of CD44⁺/CD133⁺ PCSCs increases substantially in PCa cell lines; these enriched cells exhibit enhanced tumorigenicity, invasiveness, and metastatic potential in vivo, providing direct experimental evidence that PCSCs drive therapeutic resistance, disease relapse, and distant metastasis [54, 55]. Nevertheless, conventional solid-tissue biopsies remain highly invasive, carry significant risks of complications, and suffer from sampling bias due to intratumoral heterogeneity, which can result in underestimation of Gleason grade, missed lesions, and the inability to support long-term monitoring. Liquid biopsy overcomes these limitations by enabling non‑invasive, serial assessment of circulating tumor cells (CTCs), exosomal proteins, and circulating tumor DNA (ctDNA) from peripheral blood. Clinically, CTC enumeration and surface expression profiling of PCSC‑associated markers, together with the detection of stemness‑related genetic alterations in peripheral blood, are now being validated for PCa management. A phase III prospective randomized trial in metastatic hormone‑sensitive PCa established that patients with baseline CTC counts ≥ 5 per 7.5 mL had significantly higher risks of death and disease progression, as well as markedly reduced OS, thus confirming CTC count as an independent prognostic biomarker [56]. Similarly, a prospective liquid biopsy study in mCRPC revealed aberrant overexpression of epithelial markers such as cytokeratin 19 (CK19) and prostate-specific membrane antigen (PSMA), the EMT regulator TWIST1, and the PCSC marker ALDH1 in EpCAM⁺ CTCs; their expression levels correlated strongly with poorer OS and therefore represent actionable, non‑invasive indicators for real‑time monitoring of disease evolution and treatment response [57]. Furthermore, in patients with high-risk localized PCa undergoing radical prostatectomy, an elevated fraction of CD133⁺ CTC‑derived fragments predicted a higher risk of 1‑year biochemical recurrence [58]; likewise, metastatic PCa patients with a high prevalence of CD133⁺ CTCs show accelerated progression to castration resistance following ADT, while elevated CD133 mRNA in peripheral blood mononuclear cells independently predicts shorter cumulative survival in metastatic PCa [59]. Notably, BMI1, secreted by PCa cells, enters the systemic circulation, and its serum concentration rises progressively with tumor stage and correlates positively with serum PSA levels. Importantly, BMI1 remains detectable even in patients with low PSA levels, suggesting that quantitative serum BMI1 measurement may serve as a complementary, non‑invasive diagnostic method that, when used synergistically with PSA, could improve early detection, refine prognostic assessment, and guide active surveillance [60]. Looking ahead, the integration of patient‑derived organoid models, single‑cell multi‑omics platforms, and machine learning algorithms to link PCSC‑specific molecular signatures, dynamic liquid biopsy data, and conventional clinicopathological parameters holds transformative potential for real‑time, adaptive, and personalized evaluation of PCa progression, therapeutic efficacy, and long‑term outcomes.

### Mechanisms of canonical Wnt signaling in regulating PCSCs

#### Genetic alterations of canonical Wnt signaling in PCa

The canonical Wnt signaling pathway plays a central role in PCSC formation, maintenance, and CRPC progression. Its activation directly induces transcription of stemness-associated genes, including *c-MYC*, *CD44*, *PROM1* (CD133), *LGR5*, *POU5F1*, *SOX2*, *NANOG*, *BMI1*, and *ALDH1A1*, whose elevated expression enhances PCSC self-renewal, invasiveness, and therapeutic resistance [61, 62]. Canonical Wnt pathway activation primarily arises from genomic amplifications, gain-of-function mutations, or inactivating alterations in its key components. Such alterations are significantly enriched in PCa, particularly in mCRPC, and strongly correlate with sustained PCSC stemness. In advanced PCa, approximately 20% of patients harbor genomic alterations in Wnt pathway components. A cohort study of 1,498 patients with advanced PCa identified activating Wnt pathway alterations in 193 individuals (12.9%), predominantly involving *CTNNB1* or *RSPO2* gain-of-function mutations and *APC*, *RNF43*, or *ZNRF3* loss-of-function mutations. These alterations were significantly associated with ductal features on histology, increased visceral metastasis, and co-occurring mutations in *PTEN*, *RB1*, *AR*, and *SPOP* [63]. Another study involving 4,138 PCa samples, including 3,684 microsatellite-stable (MSS) tumors, revealed that the prevalence of activating mutations in the canonical Wnt signaling pathway in MSS tumors was 10.1% in primary lesions and 19.1% in metastatic lesions; *APC* inactivation accounted for 47–54% of these activated cases. Prior studies demonstrate that genomic *APC* loss–driven canonical Wnt activation not only enhances PCa stemness and invasion via upregulation of aryl hydrocarbon receptor (AHR) and Runt-related transcription factor 1 (RUNX1) but also promotes metastatic progression through receptor tyrosine kinase-like orphan receptor 1 (ROR1) induction and Wnt5a–mediated non-canonical Wnt signaling [64]. Moreover, tumors with canonical Wnt signaling activation exhibit a higher *SPOP* mutation burden, elevated expression of canonical activators such as cystatin-1 (CST1) and odontogenic ameloblast-associated protein (ODAM), attenuated AR signaling, an immunosuppressive TME, and increased ROR1 expression, indicating synergistic crosstalk between canonical and non-canonical Wnt signaling in PCa progression [65]. Clinically, mCRPC patients with canonical Wnt–activating genetic alterations show diminished response to first-line abiraterone or enzalutamide therapy [66]. RSPO2 amplification is the most prevalent canonical Wnt pathway genetic alteration in PCa, exceeding the frequencies of *CTNNB1* mutations (9%) and *APC* loss (8%). In metastatic PCa, *RSPO2* amplification occurs in up to 22% of cases, significantly higher than *CTNNB1* mutations (9%), *APC* inactivation (8%), and *RSPO2* amplification (6.5%) in primary PCa. At the cellular level, RSPO2 overexpression, but not CTNNB1 overexpression, upregulates EMT-associated transcription factors zinc finger E-box binding homeobox 1 (ZEB1), ZEB2, and TWIST-related protein 1 (TWIST1), suggesting RSPO2 enhances the invasive capacity and malignant phenotype of PCSCs through both Wnt-dependent and Wnt-independent mechanisms. Clinical data also show that RSPO2 amplification are significantly associated with shorter disease-free survival in PCa patients [67]. Collectively, these findings indicate that aberrant activation of the canonical Wnt signaling, driven by gene amplification or mutation in its components, is a pivotal driver of PCa progression.

#### Regulation of canonical Wnt signaling by non-coding RNAs

**Fig. 2:**
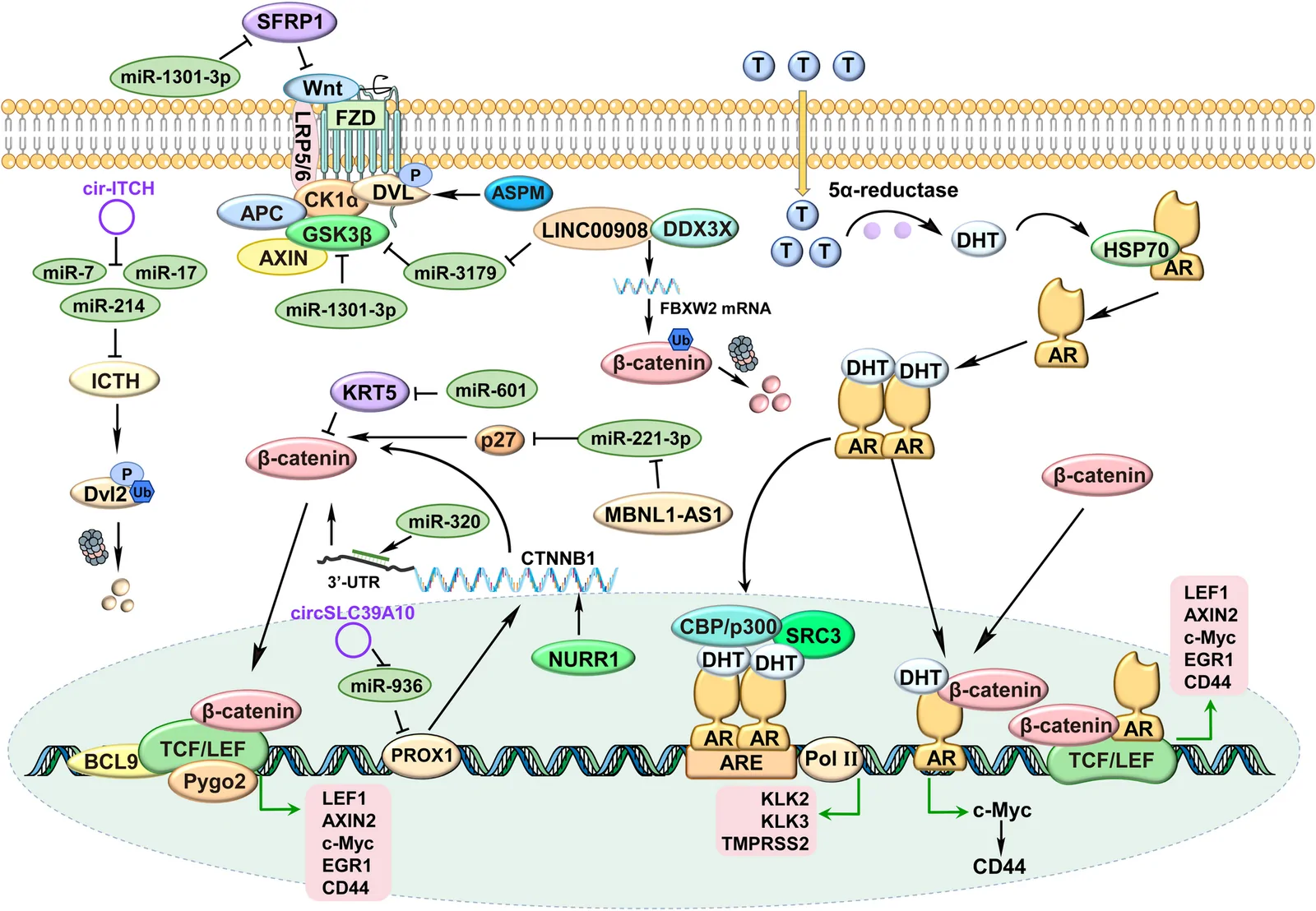
NcRNAs regulating canonical Wnt signaling, and the crosstalk between canonical Wnt and AR signaling pathways in PCSC maintenance. Endogenous ncRNAs, including miRNAs, lncRNAs, and circRNAs, regulate canonical Wnt signaling by targeting crucial signaling components, such as GSK3β, DVL, and β‑catenin, thereby affecting PCSC‑associated gene expression. In parallel, AR signaling, triggered by DHT, promotes AR nuclear translocation, driving the expression of target genes that promote PCa progression, such as KLK2/3 and TMPRSS2. The crosstalk between AR and β‑catenin leads to the co‑activation of canonical Wnt‑ or stemness‑associated target genes, including c‑MYC, CD44, LEF1, and AXIN2, which drives PCa progression and CRPC development

Non-coding RNAs (ncRNAs), including microRNAs (miRNAs), long non-coding RNAs (lncRNAs), and circular RNAs (circRNAs), serve as critical post-transcriptional regulators that modulate canonical Wnt signaling activity through diverse mechanisms, thereby influencing the function of PCSC self-renewal, stemness maintenance, and therapeutic resistance (Fig. 2). miRNAs exert post-transcriptional control over canonical Wnt signaling by binding to the 3′-untranslated regions (3′-UTRs) of mRNAs encoding pathway-related components, leading to translational repression or mRNA decay; this ultimately affects PCSC stemness and therapeutic resistance. miR-1301-3p is significantly upregulated in PCa tissues and cell lines. By directly targeting the canonical Wnt pathway repressors GSK3β and secreted frizzled-related protein 1 (SFRP1), it alleviates inhibition of canonical Wnt signaling, promotes β-catenin nuclear translocation, and upregulates stemness-associated genes, including *POU5F1*, *SOX2*, *NANOG*, *CD44*, and *MYC*, thereby enhancing PCSC self-renewal [68]. Similarly, miR-601 activates canonical Wnt signaling by suppressing its target gene *KRT5*; conversely, miR-601 knockdown reverses this effect and potently inhibits PCSC proliferation, migration, and invasion [69]. In contrast, miR-320 is significantly downregulated in PCa tissues, where it directly binds the 3′-UTR of *CTNNB1* mRNA to suppress β-catenin expression. Overexpression of miR-320 attenuates PCSC stem-like properties, whereas miR-320 depletion enhances tumor spheroid formation and chemoresistance [70].

LncRNAs and circRNAs also regulate the canonical Wnt signaling and PCSC stemness. LINC00908 is downregulated in PCa cells through transcriptional repression by the histone deacetylase 2 (HDAC2)–p300–YY1 complex. Its overexpression increases GSK3β expression by sponging miR-3179 and concurrently recruits DEAD-box helicase 3 X-linked (DDX3X) to stabilize F-box and WD repeat domain containing 2 (FBXW2) mRNA, leading to enhanced ubiquitination and degradation of β-catenin. This suppresses canonical Wnt signaling and ultimately inhibits PCSC stem-like properties and tumor growth [71]. Similarly, MBNL1-AS1 is downregulated in PCa tissues and correlates negatively with patient survival, tumor progression, and therapeutic resistance. By competitively binding miR-221-3p, MBNL1-AS1 upregulates p27 expression, thereby activating canonical Wnt signaling and promoting PCSC stemness, proliferation, and invasion [72]. Furthermore, circSLC39A10 is significantly upregulated in PCa tissues and cells and associates with multiple clinicopathological features, suggesting its potential as a diagnostic biomarker. Mechanistically, circSLC39A10 sequesters miR-936, relieving its translational repression of PROX1 mRNA; elevated prospero homeobox protein 1 (PROX1) enhances β-catenin expression and nuclear accumulation, thereby activating the canonical Wnt signaling and driving PCa cell proliferation, migration, and metastasis [73]. Conversely, cir-ITCH exerts tumor-suppressive effects by sponging miR-7, miR-17, and miR-214 to elevate itchy E3 ubiquitin protein ligase (ITCH) expression, which promotes phosphorylation and ubiquitin-mediated degradation of DVL2, thus inhibiting canonical Wnt signaling and markedly suppressing proliferation, migration, and metastasis in both hormone-sensitive PCa and CRPC cells [74, 75]. In summary, ncRNAs modulate the canonical Wnt signaling by targeting both its positive and negative regulators, thereby controlling the stemness, self-renewal, and therapy resistance of PCSCs. These findings not only deepen our understanding of epigenetic regulation in PCSCs but also reveal promising molecular targets for novel therapeutic strategies directed against ncRNAs or their downstream Wnt pathway components.

#### Epigenetic regulation of Wnt signaling

In PCa, aberrant DNA methylation of Wnt pathway components and their regulatory molecules is frequently observed, and their methylation status shows promise as a diagnostic and prognostic biomarker. For instance, SFRP2, SFRP3, and SFRP5 are transcriptionally silenced due to hypermethylation of their promoter regions, leading to diminished inhibition of canonical Wnt signaling; conversely, reduced SFRP1 expression results from both promoter hypermethylation and elevated EZH2-mediated trimethylation of histone H3 lysine 27 (H3K27me3) [76]. Overexpression of the chromatin remodeling protein high mobility group AT-hook 2 enhances histone acetylation at target loci, upregulates Wnt2 and Wnt4 expression in prostate stromal cells, and subsequently activates canonical Wnt signaling in adjacent epithelial cells via paracrine signaling, thereby promoting prostatic intraepithelial neoplasia [77]. Wnt5a overexpression is strongly associated with hypomethylation of its gene locus in PCa. In normal prostate cells, three CpG sites (CpG15, CpG24, and CpG35) within the first exon of Wnt5a are highly methylated; in contrast, site-specific demethylation occurs in cancer cells, and demethylation at CpG35 specifically restores c-Myb binding to this region, relieving transcriptional repression and driving aberrant Wnt5a overexpression [78]. Although members of the DKK family are classically defined as inhibitors of canonical Wnt signaling, they can also exert oncogenic functions in a context-dependent manner, depending on the cellular microenvironment, disease stage, and the relative activity of distinct Wnt signaling branches. For example, DKK1 is markedly overexpressed in mCRPC tissues, where it promotes tumor growth, invasion, and immune evasion, a phenotype linked to DKK1 promoter hypomethylation and concurrent activation of canonical Wnt signaling [79]. As a transcriptional coactivator of the canonical Wnt signaling, CBP/p300 binds β-catenin at its C-terminus via the TAZ1 and TAZ2 domains to drive transcription of oncogenes including c-MYC and CCND1; additionally, CBP/p300 acetylates the AR and p53, thereby facilitating malignant progression of PCa [15, 80]. Collectively, epigenetic alterations dysregulate Wnt signaling at multiple functional levels, including ligands, antagonists, and transcriptional coactivators, through mechanisms such as promoter methylation and histone post-translational modifications. Consequently, these changes may augment the stemness and self-renewal capacity of PCSCs, promoting PCa progression and therapeutic resistance. A deep understanding of these epigenetic mechanisms holds significant potential for improving prevention strategies, enabling molecular subtyping, and guiding targeted therapies, particularly for CRPC.

### Synergistic interactions between canonical Wnt signaling and other oncogenic pathways in PCa development and PCSC maintenance

The canonical Wnt signaling does not function independently during PCa progression, particularly in CRPC; rather, it engages in extensive crosstalk with other key oncogenic pathways, including AR, PI3K/AKT/mTOR, and Hippo–YAP/TAZ pathways. Through shared regulatory nodes such as GSK-3β, these pathways form an integrated, reciprocally reinforcing signaling network that drives PCa progression toward CRPC, sustains PCSC stemness, and induces therapeutic resistance. Consequently, elucidating the mechanisms underlying this pathway crosstalk is essential for designing rational combination therapies for CRPC.

#### Canonical Wnt and AR signaling pathways

The AR signaling pathway is a central regulator of prostate epithelial cell differentiation, proliferation, and survival. Upon binding of testosterone (T) or its active metabolite dihydrotestosterone (DHT) to the AR ligand-binding domain, AR dissociates from heat shock protein complexes, undergoes conformational activation, dimerizes, and translocates to the nucleus. There, it binds androgen response elements in promoter or enhancer regions of target genes and recruits coactivators and transcriptional machinery to initiate transcription of downstream genes such as kallikrein related peptidase 3 (*KLK3)*, transmembrane serine protease 2 (*TMPRSS2)*, and *KLK2*, thereby regulating the cell cycle, differentiation, and anti-apoptotic processes. Aberrant activation of this pathway is a key driver of PCa initiation and early progression. Although ADT suppresses AR signaling, compensatory reactivation occurs via multiple mechanisms, including gene amplification, mutations, splicing variants (e.g., AR-V7), intratumoral androgen synthesis, and co-regulator remodeling. These adaptations enable ligand-independent or low-ligand AR activity, sustaining tumor growth under castrate conditions. AR signaling also engages in functional crosstalk with the canonical Wnt pathway, jointly promoting the progression of PCa to CRPC (Fig. 2). In the presence of DHT, β-catenin directly interacts with the AR ligand-binding domain; the AR–β-catenin complex is recruited to regulatory regions of the *c-MYC* locus, enhancing its transcription and subsequent upregulation of CD44. This cooperative regulation promotes oncogenic transformation of prostate epithelial cells and accelerates tumor growth and invasion [81]. Moreover, AR and β-catenin are co-recruited to β-catenin/TCF4-binding sites, driving expression of stemness- and metastasis-associated target genes, including *LEF1*, *AXIN2*, *c-MYC*, early growth response 1 (*EGR1)*, and *CD44* [82]. Further studies demonstrate that in the Osr1⁺ lineage, a defined subpopulation of prostate stem cells, aberrant activation of AR signaling upregulates insulin-like growth factor 1 (IGF1) signaling, initiates oncogenic transformation, and potentiates canonical Wnt activity. This reciprocal reinforcement enhances PCSC stemness and invasive capacity, promoting PCa progression. Additionally, several positive regulators of canonical Wnt signaling contribute to PCSC maintenance. ASPM is a novel co-activator of this cascade. By directly binding DVL3, it impedes proteasomal degradation, stabilizes DVL3 protein, and thereby amplifies canonical Wnt signaling, significantly expanding the population, stemness, and tumorigenic potential of ALDH1A1⁺ PCSCs [83]. The orphan nuclear receptor NURR1 is highly expressed in PCSCs and exerts dual transcriptional activity. It directly activates *CTNNB1* transcription to elevate β-catenin levels and concurrently enhances TCF-dependent transcriptional output, resulting in upregulated expression of PCSC markers such as CD44 and NANOG. This promotes EMT, stemness maintenance, and metastasis in CRPC [84]. Collectively, these findings underscore the therapeutic rationale for combining ADT with inhibition of the canonical Wnt signaling pathway to impede PCa malignant progression.

#### Canonical Wnt and PI3K/AKT/mTOR signaling pathways

**Fig. 3:**
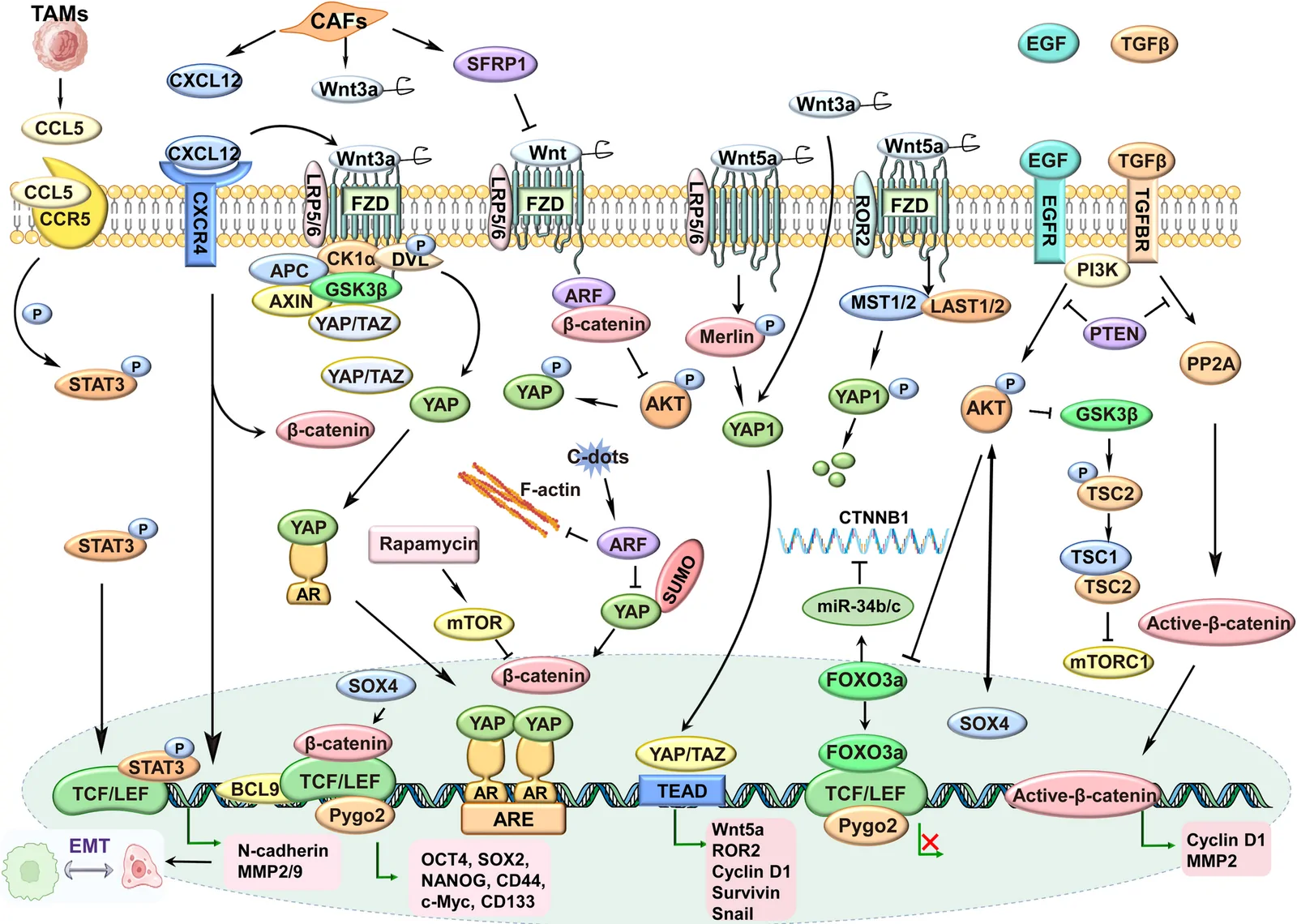
Crosstalk of canonical Wnt signaling with PI3K/Akt/mTOR and Hippo-YAP/TAZ pathways, and the TME in PCSC maintenance. PTEN inactivation drives constitutive PI3K/AKT signaling, which upregulates PP2A and leads to dephosphorylation of β-catenin. Active β-catenin accumulates in the nucleus and activates canonical Wnt signaling. AKT-mediated phosphorylation of FOXO3a triggers its nuclear exclusion and inactivation, thereby relieving its competitive inhibition of β-catenin/TCF4 and alleviating miR-34b/c–mediated repression of CTNNB1. SOX4 binds the β-catenin/TCF4 complex to amplify canonical Wnt signaling, and simultaneously sustains AKT activation while inhibiting GSK3β, thus stabilizing β‑catenin. In PCa, the inhibition of LATS1/2 kinase activity permits YAP/TAZ dephosphorylation and nuclear translocation, where they associate with TEADs to induce downstream oncogenic genes. Wnt3a promotes nuclear translocation of YAP and AR independent of β‑catenin. High ROR2 levels activate the MST1/2–LATS1 axis to suppress YAP. Activated YAP1 transcriptionally upregulates Wnt5a and ROR2, forming a negative feedback loop. ARF antagonizes this signaling by binding β‑catenin to inhibit AKT, reducing YAP phosphorylation. It also impairs YAP SUMOylation, blocking its nuclear translocation and attenuating F-actin assembly, thereby inhibiting CRPC metastasis. Tea‑derived carbon dots (CDs) reduce nuclear accumulation of YAP and β‑catenin. In the prostate TME, CAFs and TAMs promote PCSC maintenance, migration, invasion, and malignant progression through paracrine networks. CAFs can not only secrete SFRP1 to competitively bind Wnt ligands and promote the canonical Wnt signaling, but also secrete ligands such as CXCL12 and Wnt3a to activate the CXCL12/CXCR4 axis and canonical Wnt signaling, respectively, thus upregulating the expression of CSC‑related genes, including OCT4, SOX2, NANOG, CD44, c‑Myc, and CD133. TAMs secrete CCL5 to activate the CCR5/β‑catenin/STAT3 signaling pathway, thereby inducing expression of EMT-associated markers, such as N‑cadherin and MMP2/9, and augmenting PCSC invasiveness

The PI3K/AKT/mTOR signaling pathway governs fundamental cellular processes including cell growth, survival, protein synthesis, glucose metabolism, and autophagy [85]. Under physiological conditions, its activation is precisely controlled by multilayered negative feedback mechanisms to ensure signal fidelity and preserve cellular homeostasis. In PCa, however, loss-of-function alterations in key negative regulators such as PTEN and TSC1/TSC2, coupled with gain-of-function events in positive regulators such as PIK3CA, AKT1, and PIK3R1, result in consistent pathway hyperactivation. This aberrant signaling cooperates with the canonical Wnt signaling to drive cancer progression and confer resistance to ADT and/or PI3K/AKT/mTOR inhibitors (Fig. 3) [86–89]. PTEN loss-induced sustained PI3K pathway activation in PCa cells upregulates protein phosphatase protein phosphatase 2A (PP2A), which dephosphorylates β-catenin at N-terminal residues Ser37 and Thr41, generating active β-catenin and facilitating its nuclear translocation to activate transcription of target genes, including CCND1 and matrix metallopeptidase 2 (MMP2). Unlike the canonical Wnt signaling, which controls β-catenin protein stability through the destruction complex, the PTEN/PI3K/PP2A axis specifically governs the generation and subcellular redistribution of active β-catenin [90]. Moreover, the PI3K/AKT/mTOR pathway indirectly modulates β-catenin transcriptional activity through downstream effectors forkhead box O3 (FOXO3a) and SOX4. The tumor suppressor FOXO3a antagonizes canonical Wnt signaling by two mechanisms: (i) competing with TCF4 for binding to β-catenin, thereby blocking transcriptional complex assembly; and (ii) transactivating miR-34b/c to suppress *CTNNB1* mRNA expression. Conversely, PI3K/AKT-mediated phosphorylation of FOXO3a triggers its nuclear exclusion and functional inactivation, relieving inhibition of Wnt target gene transcription and promoting EMT and metastasis in PCa [91]. In contrast, the oncogenic transcription factor SOX4 establishes a reciprocal positive feedback loop with both pathways. As a direct transcriptional target of β-catenin/TCF, SOX4 protein enhances canonical Wnt signaling by stabilizing the β-catenin/TCF complex; simultaneously, PTEN loss-driven PI3K/AKT/mTOR activation induces SOX4 expression, while SOX4 reciprocally sustains AKT phosphorylation and inhibits GSK3β activity, thereby stabilizing β-catenin and further amplifying canonical Wnt signaling. Collectively, this bidirectional crosstalk accelerates PCa progression and metastasis [92]. Additionally, GSK3β and TSC1/TSC2 serve as critical molecular nodes bridging these pathways. The TSC1/TSC2 complex functions as a major endogenous inhibitor of mTORC1 [93]. Upon canonical Wnt signaling activation, GSK3β inhibition reduces inhibitory phosphorylation of TSC2, destabilizing the TSC1/TSC2 complex and consequently derepressing mTORC1, leading to its hyperactivation, enhanced proliferative capacity, and promotion of CRPC [94]. Conversely, the LRP6 inhibitor salinomycin suppresses PCa cell proliferation and induces apoptosis by reactivating GSK3β, thereby attenuating the co-activation of cyclin D1 and survivin by both canonical Wnt and PI3K/AKT/mTOR signaling. In summary, the crosstalk between the PI3K/AKT/mTOR and canonical Wnt signaling pathways constitutes a central driver of aggressive PCa progression; deciphering their mechanistic interplay offers a rational foundation for overcoming therapeutic resistance to ADT and PI3K/AKT/mTOR inhibitors.

#### Canonical Wnt and Hippo–YAP/TAZ signaling pathways

The Hippo signaling pathway is an evolutionarily conserved kinase cascade critically involved in organ development, tissue homeostasis, regeneration, and immune regulation, with its core biological effects mediated by the downstream transcriptional coactivators YAP and TAZ. Under physiological conditions, the core kinases macrophage stimulating 1/2 (MST1/2) and large tumor suppressor kinase 1 (LATS1/2) sequentially phosphorylate YAP and TAZ, triggering their cytoplasmic retention and ubiquitin-mediated proteasomal degradation, thereby suppressing their nuclear transcriptional activity [95–97]. In PCa, dysregulation of upstream regulators, such as loss of cell–cell contact inhibition, leads to diminished LATS1/2 kinase activity. Consequently, YAP/TAZ undergo sustained dephosphorylation, accumulate in the nucleus, and bind TEAD transcription factors to drive expression of oncogenic targets such as *CCND1* and *SNAI1* (Snail), thereby promoting malignant proliferation, evasion of apoptosis, EMT, maintenance of PCSC properties, and ADT resistance. Critically, hyperactivated YAP/TAZ engages in reciprocal positive crosstalk with the canonical Wnt signaling, accelerating cancer progression and therapeutic resistance. Evidence indicates that YAP/TAZ functions not only as a key downstream effector of canonical Wnt signaling but also as a bidirectional regulator of this pathway [98, 99]. Upon Wnt activation, YAP/TAZ dissociates from inhibitory complexes and translocates to the nucleus to induce target gene transcription. However, in PCa cells under ADT, Wnt3a induces nuclear translocation of both YAP and the AR to drive oncogenic transcription, without requiring β-catenin nuclear accumulation, indicating a non-canonical, β-catenin–independent mechanism of Wnt3a action [100]. Furthermore, non-canonical Wnt signaling exerts context-dependent regulation of YAP. In ROR2-high PCa cells, Wnt5a activates MST1/2–LATS1 signaling to phosphorylate YAP1 at Ser127, promoting its cytoplasmic sequestration and exerting tumor-suppressive effects; conversely, in ROR2-deficient cells, Wnt5a induces Merlin phosphorylation at Ser518, leading to YAP1 activation and pro-tumorigenic outcomes. Notably, activated YAP1 transcriptionally upregulates Wnt5a and its receptor ROR2, establishing a self-reinforcing loop that inhibits the overactivation of YAP1 signaling [101]. Moreover, ARF, the alternative reading frame protein product of the cyclin-dependent kinase inhibitor 2 A, acts as a tumor suppressor and coordinately regulates both the Hippo and canonical Wnt signaling pathways through two distinct mechanisms. On one hand, ARF binds β-catenin and inhibits AKT, which reduces the inhibitory phosphorylation of YAP at Ser127, stabilizes β-catenin, and promotes cytoplasmic retention of YAP, thereby restricting its oncogenic transcriptional activity. On the other hand, ARF impairs YAP SUMOylation, blocking its nuclear translocation and attenuating F-actin assembly, which preserves the tumor-suppressive function of cytoplasmic YAP and inhibits the migration of CRPC cells. Leveraging this mechanism, the combination of tea-derived carbon dots, which stimulate ARF expression, with rapamycin-mediated mTOR inhibition synergistically reduces the nuclear localization of both YAP and β-catenin, thereby effectively suppressing the proliferation and migration of CRPC cells and offering a novel combinatorial strategy against therapeutic resistance [102]. In summary, crosstalk between the Hippo–YAP/TAZ and canonical Wnt signaling pathways drives PCa aggressiveness and treatment resistance (Fig. 3), offering a mechanistic basis for rational combination therapies targeting CRPC.

#### Canonical Wnt signaling and metabolic reprogramming

Metabolic reprogramming not only drives PCa initiation and progression but also critically sustains PCSC stemness, therapeutic resistance, and metastasis. Unlike differentiated tumor cells, which predominantly rely on aerobic glycolysis, PCSCs exhibit high metabolic plasticity and preferentially utilize mitochondrial oxidative phosphorylation and fatty acid oxidation to meet energetic demands and maintain self-renewal. This metabolic adaptation enables PCSCs to survive in hypoxic and nutrient-deprived microenvironments, thereby evading the cytotoxic effects of radiotherapy, chemotherapy, and ADT [103, 104]. The canonical Wnt signaling and metabolic reprogramming reciprocally regulate each other engage and jointly drive PCa progression and therapeutic resistance. For example, radiation-resistant PCa cells and PCSCs both display heightened dependence on glutaminase (GLS)-mediated glutamine catabolism to maintain redox homeostasis and α-ketoglutarate-dependent epigenetic regulation. This dependence is mediated by the canonical Wnt signaling, which transcriptionally represses miR-23a/b via its target gene c-MYC, leading to significant upregulation of mitochondrial GLS expression. Elevated c-Myc and GLS levels correlate significantly with shorter progression-free survival after radiotherapy, and pharmacological inhibition of GLS or c-Myc reduces PCSC frequency and restores radiosensitivity [105, 106]. Moreover, canonical Wnt signaling increases mitochondrial biogenesis by upregulating transcription factors such as c-Myc and SOX2, enhances glycolysis and oxidative phosphorylation, and promotes lineage plasticity in PCa cells, thereby facilitating tumor progression, metastasis, and therapeutic resistance [107, 108]. Concurrently, sustained aerobic glycolysis leads to intracellular lactate accumulation; lactate serves as a substrate for β-catenin lactylation, which enhances β-catenin stability and further amplifies canonical Wnt signaling [109]. In PTEN/p53-deficient PCa, persistent canonical Wnt activation induces cancer cells to secrete abundant lactate, which lactylates histone H3K18 in tumor-associated macrophages (TAMs). This modification activates transcription of M2-polarization genes, driving TAMs toward an immunosuppressive and pro-angiogenic phenotype and thus promoting immune evasion and tumor progression. Accordingly, combined inhibition of PI3K/MEK and the canonical Wnt signaling with LGK974 achieves durable tumor control by suppressing H3K18 lactylation and restoring TAM phagocytic function [110].

Compared with most solid tumors, PCa exhibits a greater reliance on lipid metabolism, and reciprocal crosstalk between Wnt signaling pathways and lipid-metabolizing enzymes underpins this dependency. Fatty acid synthase (FASN) activates canonical Wnt signaling by promoting Wnt1 palmitoylation, thereby stimulating PCa cell proliferation and anti-apoptotic signaling [111]. Conversely, the non-canonical ligand Wnt5a upregulates free cholesterol levels in PCa cells, leading to AR activation and sustained growth under androgen-deprived conditions, which contributes to ADT resistance [112]. Furthermore, cholesterol esters accumulation is consistently observed in PCa metastatic lesions; inhibition of cholesterol esterification reduces monounsaturated fatty acid availability, thereby impairing Wnt3a secretion and acetylation and suppressing Wnt-driven metastasis [113]. These findings underscore the synergistic contribution of lipid metabolism and Wnt signaling to PCSC stemness maintenance during PCa progression. In summary, the interplay between aberrant canonical Wnt signaling and metabolic reprogramming cooperatively shape the aggressive phenotypes of PCa. Targeting PCSC-specific vulnerabilities, particularly in oxidative phosphorylation and lipid metabolism, represents a promising strategy for the precise eradication of PCSCs.

#### Canonical Wnt signaling and the prostate TME

The formation and maintenance of PCSCs depend not only on intrinsic signaling networks within tumor cells but also on extrinsic cues from cellular components and secreted factors in the TME. Among these, CAFs and TAMs promote PCSC stemness, self-renewal, and proliferative capacity by secreting canonical Wnt ligands and modulators (Fig. 3) [114–116]. CAFs constitute a pivotal stromal component that regulates PCSC behavior. Evidence indicates that CAFs activate the canonical Wnt signaling through multiple mechanisms, thereby augmenting PCSC stemness and proliferation [114]. SFRP1, a secreted Wnt antagonist, normally inhibits canonical Wnt signaling by using its cysteine-rich domain to sequester Wnt ligands and block their binding to FZDs. Paradoxically, in PCa, CAF-derived SFRP1 acts in a paracrine manner to activate canonical Wnt signaling in cancer cells, upregulating CSC-related genes such as *SOX2*, *NANOG*, *POU5F1*, and *c-MYC*, and increasing both the frequency and invasive potential of PCSCs [115]. Moreover, CAFs overexpress and secrete Wnt3a and C-X-C motif chemokine ligand 12 (CXCL12), cooperatively activating the canonical Wnt and CXCL12/C-X-C motif chemokine receptor 4 (CXCR4) pathways to elevate expression of stemness markers CD133 and CD44, thereby reinforcing PCSC stemness. Notably, inhibition of the canonical Wnt signaling does not impair CXCL12/CXCR4 signaling, whereas blockade of CXCL12/CXCR4 significantly suppresses canonical Wnt activity, indicating that CXCL12/CXCR4 signaling functionally precedes and enables Wnt pathway activation in this context [114]. TAMs represent the most abundant immune cell population in the TME. TAM-secreted C-C motif chemokine ligand 5 (CCL5) activates both C-C motif chemokine receptor 5 (CCR5)/β-catenin and STAT3 signaling in PCSCs, markedly enhancing their functional activity and self-renewal. Concurrently, CCL5 induces expression of EMT markers, including N-cadherin, MMP2, and MMP9, thereby augmenting PCSC invasiveness [116].

### Canonical Wnt signaling drives EMT of PCSCs

EMT is a fundamental developmental program co-opted by tumor cells to acquire invasive and metastatic capabilities. During EMT, polarized epithelial cells progressively lose cell–cell adhesion and apicobasal polarity, adopting a motile, mesenchymal phenotype characterized by enhanced migratory and invasive properties. Molecular hallmarks include downregulation of the epithelial adhesion molecule E-cadherin and upregulation of mesenchymal markers, including N-cadherin, vimentin, and fibronectin [117]. EMT is orchestrated by multiple conserved signaling pathways, notably receptor tyrosine kinase (RTK), TGF-β, Hedgehog, and canonical Wnt cascades. Among these, hyperactivation of the canonical Wnt signaling directly drives the expression of a series of EMT-related genes, including *SNAI1* (Snail), *SNAI2* (Slug), *ZEB1*, *ZEB2*, *TWIST1*, *BOP1*, cyclin-dependent kinase regulatory subunit 2 *(CKS2)*, and nuclear factor interleukin-3-regulated protein *(NFIL3)*, thereby initiating and sustaining the EMT program [118]. Critically, EMT not only confers motility and invasiveness but also promotes acquisition of CSC-like properties. Moreover, EMT-transformed cells secrete cytokines such as TGF-β and IL-6, recruit myeloid-derived suppressor cells (MDSCs) and TAMs, and activate CAFs. This establishes an immunosuppressive, pro-metastatic TME. In PCa, disease recurrence, metastasis, and treatment failure are closely associated with the convergence of CSC traits and EMT. During PCa progression, canonical Wnt hyperactivation drives EMT in cancer cells, enhancing their metastatic propensity and facilitating acquisition of PCSC-like properties. Conversely, inhibiting this cascade markedly attenuates EMT markers and functional phenotypes.

**Fig. 4:**
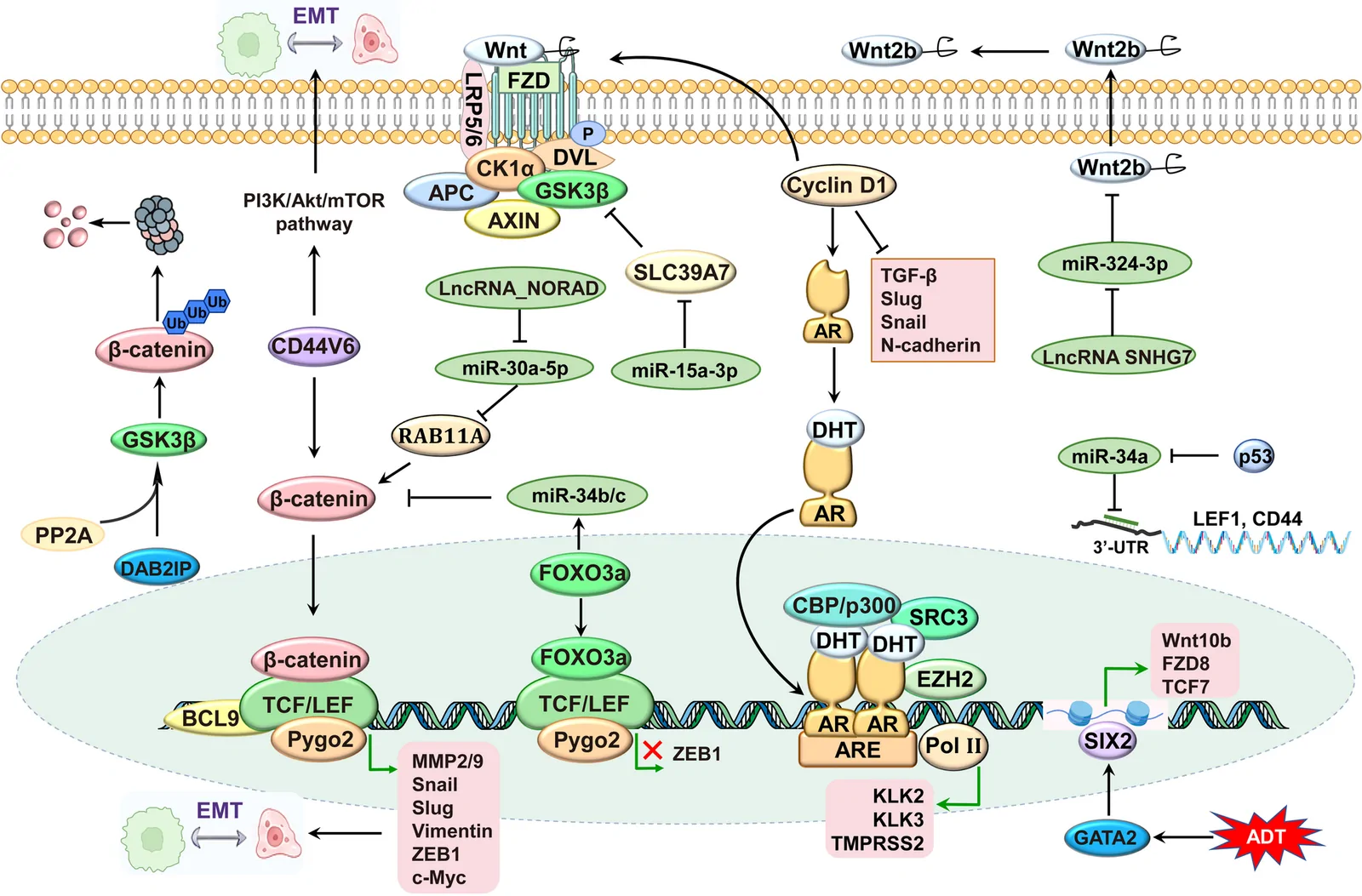
Canonical Wnt signaling promotes EMT of PCSCs. Upon pathway activation, β‑catenin translocates to the nucleus and associates with TCF/LEF to initiate transcription of EMT‑related genes. Endogenous non‑coding RNAs (miRNAs, lncRNAs, and circRNAs) modulate canonical Wnt signaling by targeting core signaling components. Cyclin D1 not only amplifies DHT‑driven AR signaling and androgen‑responsive gene expression, but also establishes a positive feedback loop that potentiates canonical Wnt activity, leading to upregulation of stemness‑associated factors and promoting PCSC expansion, while inversely suppressing some EMT‑related effectors, including TGF‑β, Snail, N‑cadherin, and Slug. CD44 variant 6 (CD44v6) activates the PI3K/Akt/mTOR and canonical Wnt signaling pathways, thereby impairing PCa cell invasiveness and tumor sphere‑forming capacity

Multiple signaling molecules and pathways modulate both EMT and PCSC stemness through canonical Wnt signaling (Fig. 4). For instance, disabled homolog 2-interacting protein (DAB2IP) recruits PP2A to activate GSK3β, thereby promoting phosphorylation and ubiquitin-mediated degradation of β-catenin, suppressing β-catenin/TCF transcriptional activity, and inhibiting EMT in metastatic PCa cells. However, epigenetic silencing of *DAB2IP*, due to promoter DNA hypermethylation and/or repressive histone modifications, is frequent in PCa, leading to loss of its canonical Wnt signaling–inhibitory function. This derepression enables β-catenin stabilization, upregulates CD44 expression, drives transformation of normal prostate epithelium toward a PCSC state, enhances chemoresistance, and cooperatively induces EMT to facilitate metastasis. Consequently, *DAB2IP* loss is a recurrent feature of metastatic PCa [119, 120]. Notably, Cyclin D1, a direct transcriptional target of β-catenin/TCF, exhibits elevated expression in PCSCs. Beyond amplifying DHT-induced proliferation and modulating AR signaling, Cyclin D1 reinforces canonical Wnt signaling via a positive feedback loop, upregulating stemness-associated genes, including SOX2 and NANOG, and expanding the PCSC population. Paradoxically, it concurrently suppresses key EMT effectors, including TGF-β, Snail, N-cadherin, and Slug [121]. Furthermore, radioresistant PCa cells exhibit enhanced PCSC features, including elevated expression of CSC markers (CD133, CXCR4, ABCG2, OCT4, NANOG), high aldehyde dehydrogenase (ALDH) enzymatic activity, EMT induction, and enhanced DNA repair capacity, with ALDH⁺ cells being particularly enriched and mechanistically coupled to canonical Wnt signaling hyperactivation. Inhibition of the canonical Wnt signaling pathway significantly reduces the number of ALDH⁺ cells, enhances their sensitivity to radiotherapy, and reduces their metastatic potential [122]. Similarly, CD44 variant 6 (CD44v6) is overexpressed in primary PCa tissues and metastatic lymph nodes; CD44v6⁺ cells exhibit PCSC features. CD44v6 knockdown simultaneously suppresses PI3K/Akt/mTOR signaling, canonical Wnt signaling, and EMT, reducing invasion, impairing tumor sphere formation, and sensitizing cells to radiotherapy and chemotherapy. These findings indicate that CD44v6 serves as a critical node integrating the PI3K/Akt/mTOR, canonical Wnt pathways, and EMT to jointly drive PCa metastasis and therapeutic resistance [123].

**Fig. 5:**
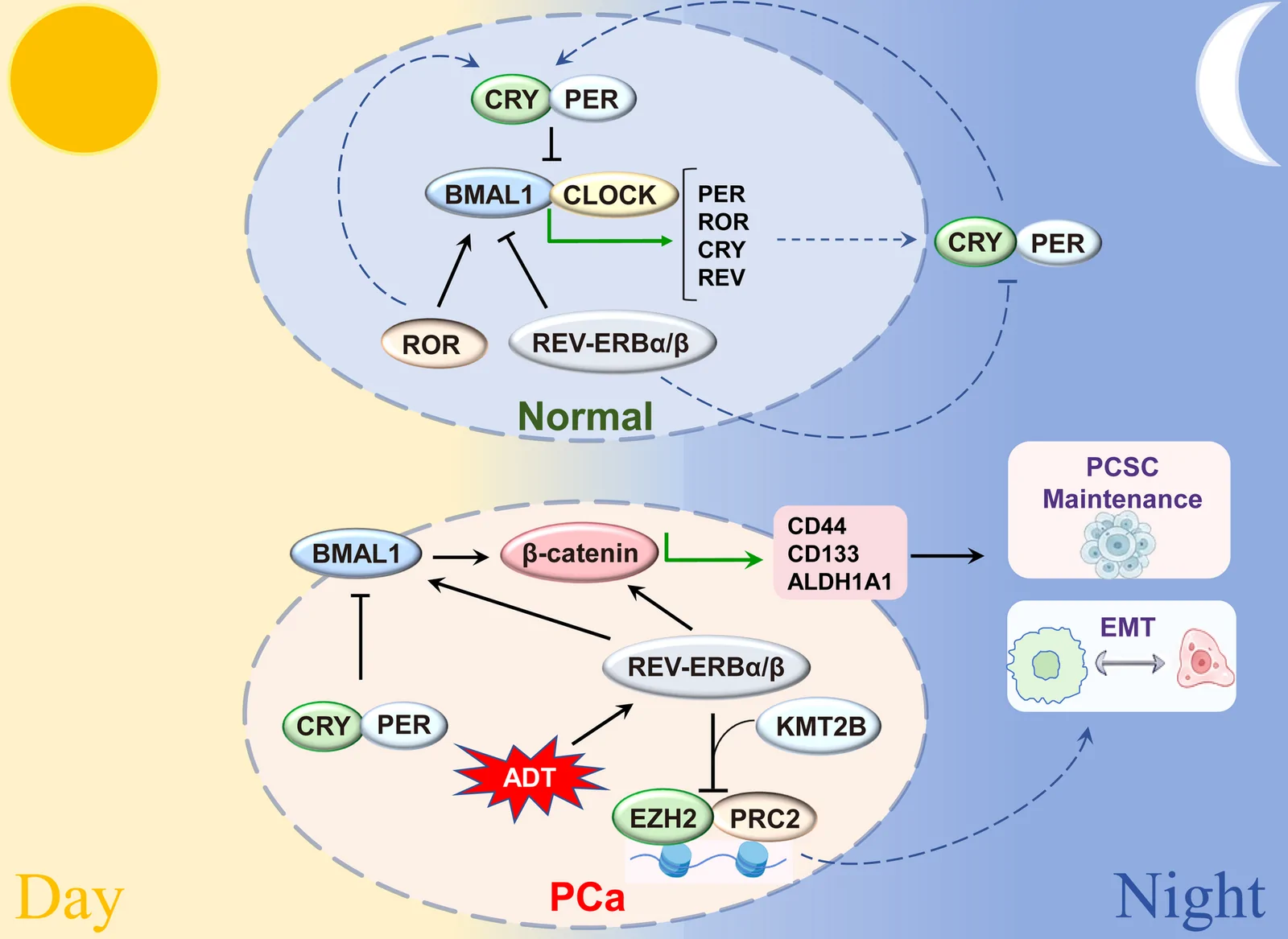
Circadian rhythm disruption promotes PCa progression via activating canonical Wnt signaling. The circadian clock is orchestrated by the CLOCK/BMAL1 activator complex, which drives PER/CRY expression, while REV‑ERBα/β and ROR act as reciprocal repressors and activators to stabilize this oscillatory system. In PCa, REV‑ERBα switches to a tumor‑promoting activator, and cooperates with canonical Wnt signaling to recruit KMT2B, counteract PRC2/EZH2 occupancy, and remodel chromatin bivalency, thereby driving PCa progression and metastasis. BMAL1 facilitates β‑catenin nuclear translocation to drive stemness‑associated gene expression

Circadian rhythm disruption is strongly associated with PCa initiation, progression, and therapeutic resistance (Fig. 5). Under physiological conditions, the circadian clock is governed by a core transcription–translation negative feedback loop. The activators CLOCK and basic helix-loop-helix ARNT like 1 (BMAL1) drive transcription of the period circadian regulator (PER) and cryptochrome circadian regulator (CRY) genes; subsequently, accumulated PER/CRY protein complexes translocate to the nucleus and repress CLOCK/BMAL1 transcriptional activity. Upon PER/CRY degradation, repression is relieved, enabling the cycle to recur with near-24-hour periodicity. Additional regulatory layers are provided by the nuclear receptors REV-ERBα/β (transcriptional repressors) and ROR (transcriptional activator), which competitively bind RORE elements in the BMAL1 promoter to fine-tune its expression and reinforce circadian amplitude and precision. Accumulating evidence indicates that REV-ERBα acquires oncogenic functions in advanced PCa, undergoing a functional switch from a canonical transcriptional repressor to a context-dependent transcriptional coactivator. It not only activates oncogenic MAPK and PI3K/Akt signaling but also cooperates with the canonical Wnt signaling to recruit the histone methyltransferase lysine methyltransferase 2B (KMT2B) and antagonize polycomb repressive complex 2 (PRC2)/enhancer of zeste homolog 2 (EZH2) occupancy, thereby activating diverse transcriptional programs that drive PCa initiation, progression, therapy resistance, and metastasis [124]. As an AR coactivator, EZH2 enhances transcription of AR target genes. However, clinical responses to PRC2/EZH2 inhibitors remain limited in PCa, potentially due to either inhibitor-induced functional reprogramming of REV-ERBα upon PRC2/EZH2 inhibition or combined ADT, both of which can trigger diverse cell state programs (including stemness and EMT), thereby exacerbating therapeutic resistance and metastatic dissemination. In contrast, dual inhibition of the canonical Wnt signaling and EZH2 restores chromatin bivalency, suppresses PCa cell state diversity, and attenuates metastasis [125]. Furthermore, the circadian regulator gene period circadian regulator 3 (PER3) is downregulated in PCSCs. Reduced PER3 expression derepresses BMAL1, promotes nuclear accumulation of β-catenin, and consequently upregulates stemness-associated genes such as ALDH1A1, CD133, and CD44, enhancing PCSC self-renewal, tumor sphere formation, and EMT [126].

During the development of ADT–induced PCa progression, the developmental transcription factor SIX homeobox 2 (SIX2) promotes EMT and therapeutic resistance by driving tumor cell lineage plasticity (Fig. 4). Specifically, AR inhibition increases chromatin accessibility at genomic loci containing SIX2 binding motifs and concurrently activates the transcription factor GATA2, which directly upregulates SIX2 transcription. As a key regulator of the stem cell transcriptional program, SIX2 directly initiates the transcription of genes associated with the canonical Wnt signaling pathway, such as Wnt10b, FZD8, and TCF7, thereby activating this cascade. This leads to upregulation of stemness-associated marker (e.g., c-Myc, SOX2, NANOG, CD44) and EMT-related effectors (e.g., MMP7), as well as increased expression of mesenchymal proteins (Snail, Slug, Vimentin), collectively enhancing PCa cell stemness, spheroid formation, and invasive capacity. In contrast, SIX2 knockdown reverses these phenotypes by disrupting this regulatory axis. Thus, SIX2 functions as a core early driver of the ADT-acquired drug-resistant phenotype in PCa; pharmacological or genetic targeting of SIX2 effectively restores lineage fidelity, suppresses metastasis, and re-sensitizes tumors to therapy [127].

In addition, certain ncRNAs modulate PCSC activity and metastatic potential by targeting key components of the canonical Wnt signaling. For instance, the tumor suppressor p53 directly transactivates miR-34a. Mature miR-34a binds to the 3′-UTRs of LEF1 and CD44 mRNAs, repressing their expression and thereby inhibiting PCSC self-renewal, tumor sphere formation, and metastatic dissemination [128, 129]. Notably, p53 inactivation frequency increases from ~ 4% in localized PCa to 40–50% in CRPC; this loss attenuates miR-34a–mediated suppression of canonical Wnt signaling, enhancing PCSC clonal expansion, regenerative capacity, and metastatic capacity. Similarly, FOXO3a induces miR-34b/c transcription to downregulate β-catenin and concurrently competes with TCF for β-catenin binding sites on target gene promoters, thereby suppressing ZEB1 expression and reducing tumor grade and metastatic spread [91]. miR-15a-3p targets SLC39A7 mRNA, impairing its activation of the canonical Wnt signaling and downregulating EMT-associated genes such as c-MYC and vimentin, resulting in suppressed PCa cell invasion and metastasis [130]. In contrast, the lncRNA SNHG7 sequesters miR-324-3p, relieving its repression of Wnt2b and consequently amplifying canonical Wnt signaling; this upregulates MMP2/9, snail, slug, and vimentin, promoting metastatic progression [131]. Similarly, lncRNA_NORAD sponges miR-30a-5p, derepressing RAB11A expression and activating canonical Wnt signaling to drive PCa proliferation, invasion, and EMT [132]. Collectively, these findings provide new potential therapeutic targets for suppressing PCa progression and metastasis

### Canonical Wnt signaling drives bone metastasis of PCSCs

**Fig. 6:**
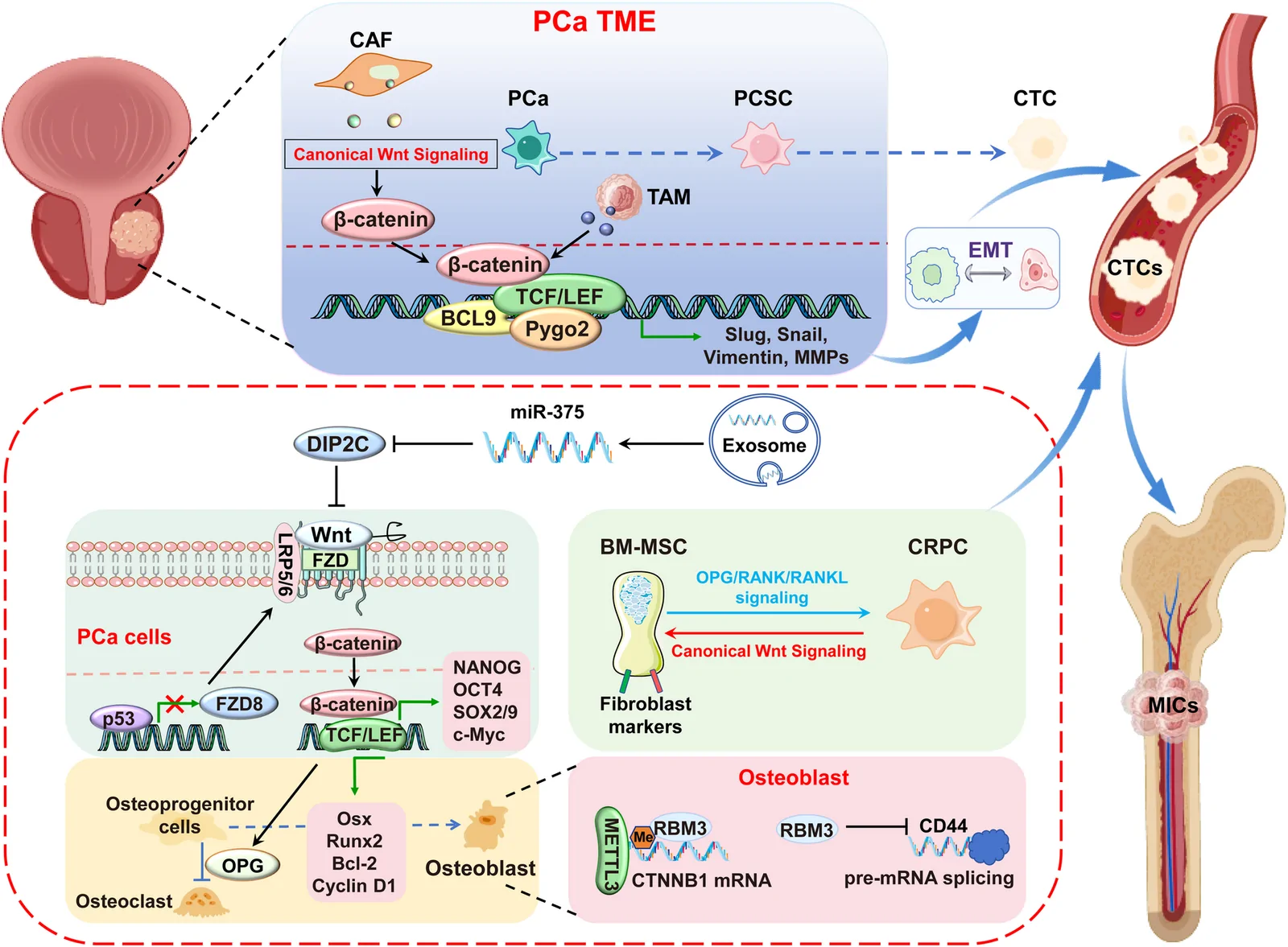
Canonical Wnt signaling drives bone metastasis of PCSCs. Canonical Wnt signaling activation, along with TME reprogramming, promotes PCSC generation and CTC dissemination. CTCs home to bone, acquire MIC traits, and initiate metastatic outgrowth. In the bone niche, canonical Wnt signaling promotes osteoblast differentiation via Runx2/Osx and suppresses osteoclast activity via OPG, while exosome‑derived miR‑375 activates canonical Wnt signaling to enhance PCa cell stemness and to induce osteogenic differentiation, driving both cancer progression and osteoblastic metastasis. The activation of OPG/RANK/RANKL signaling in bone marrow‑derived MSCs (BM‑MSCs) enhances the invasive capacity of CRPC cells, and canonical Wnt signaling activation in CRPC cells induces BM‑MSCs to express CAF markers.

Bone metastasis is the most prevalent metastatic manifestation of PCa and represents the principal clinical challenge in advanced stages. Skeletal-related complications arising from bone metastasis are the leading contributors to marked deterioration in patients’ quality of life and increased mortality. Among PCa patients who die from the disease, approximately 90% exhibit bone metastasis [133]. Accumulating evidence indicates that PCSCs serve as the initiating “seed” population for bone metastasis and exert a central role in its onset, progression, recurrence, and adverse prognosis through enhancement of migratory and invasive capacities, modulation of cellular dormancy and reactivation, and active remodeling of the bone microenvironment. During early-stage of bone metastasis, activation of the canonical Wnt signaling, induction of EMT, and dynamic remodeling of the TME collectively promote the acquisition of stem-like properties by a subset of PCa cells, thereby generating PCSCs. A fraction of these PCSCs enter systemic circulation as circulating tumor cells (CTCs) and, under guidance by chemokines, bone matrix components, and osteogenic niche signals, colonize bone tissue. Upon colonization, they further acquire functional attributes characteristic of metastasis-initiating cells (MICs), enabling establishment of metastatic lesions. Furthermore, a self-amplifying positive feedback loop progressively develops between tumor cells and the bone microenvironment, driving sustained expansion of metastatic deposits. The canonical Wnt signaling pathway regulates multiple facets of this pathological process (Fig. 6) [134].

Under physiological conditions, the canonical Wnt signaling drives the directed differentiation of MSCs into osteoblasts by upregulating key transcription factors such as Runx2 and Osterix (Osx). Concurrently, it enhances the proliferation of osteoprogenitor cells and extends the functional lifespan of mature osteoblasts through upregulation of Cyclin D1 and Bcl-2, thereby ensuring a sustained cellular supply for bone formation. Moreover, the cascade upregulates osteoprotegerin (OPG), which suppresses osteoclastogenesis and bone resorption. These mechanisms collectively promote net bone formation and increase bone mass. Consequently, pharmacological activation of canonical Wnt signaling, using agents such as GSK3β inhibitors or anti-sclerostin antibodies, is regarded as a promising therapeutic strategy for bone loss disorders including osteoporosis. However, this approach warrants caution in PCa, where aberrant pathway activation may augment cancer cell stemness and promote disease progression. For instance, the GSK3β inhibitor AR79 stabilizes β-catenin by inhibiting its phosphorylation-dependent degradation, thereby activating canonical Wnt signaling to stimulate bone formation; yet it concurrently expands the PCSC population and accelerates PCa growth [135]. PCa cells also remodel the bone microenvironment via exosome-mediated intercellular crosstalk. Notably, exosomal miR-375 selectively targets and represses DIP2C, leading to derepression and activation of the canonical Wnt signaling. This dual action enhances PCa cell stemness while inducing osteogenic differentiation of bone marrow–derived MSCs, thereby synergistically driving both cancer progression and osteoblastic metastasis. In clinical PCa specimens with bone metastasis, expression levels of miR-375, β-catenin, TCF1, and LEF1 are significantly elevated [136]. Similarly, FZD8 is markedly overexpressed in PCa cell lines and bone-metastatic tissues, and its expression positively correlates with clinical tumor stage and the burden of bone metastasis. FZD8 upregulation promotes bone metastasis by activating canonical Wnt signaling, which in turn elevates expression of PCSC stemness-related markers such as NANOG, OCT4, SOX2, SOX9, and c-Myc. In contrast, wild-type p53 suppresses FZD8 transcription and antagonizes this pro-metastatic cascade [137]. Conversely, inhibition of LRP5 significantly attenuates canonical Wnt signaling activity, thereby suppressing EMT and bone metastasis [138]. Epigenetic regulation further contributes to PCSC maintenance and canonical Wnt signaling-driven bone metastasis. Previous studies demonstrate that RNA-binding motif 3 (RBM3), a cold-stress response protein, exerts dual tumor-suppressive effects by impairing CD44 pre-mRNA splicing to diminish PCa stemness and by recruiting methyltransferase METTL3 to deposit m⁶A modifications within the 3′-UTR of *CTNNB1* mRNA, thereby reducing transcript stability, lowering β-catenin protein abundance, and dampening canonical Wnt signaling. As a result, RBM3 blunts osteoblast-induced canonical Wnt signaling activation and disrupts the reciprocal reinforcement between the bone niche and PCSCs, ultimately inhibiting PCa bone metastasis [139, 140].

The canonical Wnt signaling pathway also contributes to bone metastasis in PCa by strengthening crosstalk between bone marrow stromal cells and PCa cells. For instance, activation of the OPG/receptor activator of nuclear factor-κB (RANK)/RANK ligand (RANKL) axis in bone marrow–derived MSCs enhances the invasive capacity of CRPC cells; conversely, canonical Wnt signaling activation in CRPC cells induces bone marrow–derived MSCs to adopt a CAF-like phenotype, evidenced by upregulated expression of CAF markers, thereby further promoting bone metastasis [141]. Moreover, elevated Wnt5a expression promotes CRPC cell migration toward bone marrow stromal cell–conditioned medium, indicating its potential role in augmenting the bone-tropic capacity of PCa cells [142]; however, whether this effect is mediated through canonical Wnt signaling remains undetermined. Collectively, these findings underscore the therapeutic potential of targeting bone marrow stromal cells, including bone marrow–derived MSCs, in the bone microenvironment. In summary, the canonical Wnt signaling pathway drives PCa bone metastasis by reinforcing PCSC stemness, mediating exosome-dependent intercellular communication, and orchestrating cancer–stromal crosstalk within the bone metastatic niche; consequently, pathway-specific inhibition constitutes a highly promising therapeutic strategy for mitigating bone metastasis.

### Targeting canonical Wnt signaling for PCSC inhibition: applications, challenges and opportunities

#### Applications of targeting Wnt signaling for PCSC inhibition

Given the central role of the canonical Wnt signaling pathway in sustaining PCSC stemness, EMT, and bone metastasis, its targeted inhibition has emerged as a pivotal therapeutic strategy in PCa research. Interventions directed at distinct nodes of this pathway have demonstrated efficacy in suppressing PCSC self-renewal and stem-like properties. Here, we only discuss relevant preclinical and clinical studies with potential therapeutic value for PCSCs (Fig. 1). ETC-159, a selective Porcupine (PORCN) inhibitor, structurally mimics the palmitoylation motif of Wnt ligands and competitively occupies the PORCN active site. This blockade prevents Wnt ligands palmitoylation, leading to intracellular retention, impaired secretion, and consequent abrogation of pathway activation. Balk et al. reported that ETC-159 not only downregulates canonical Wnt signaling–driven PCSC markers AHR and RUNX1, but also suppresses expression of the non-canonical Wnt receptor ROR1, thereby concurrently inhibiting Wnt5a/ROR1-mediated signaling and significantly impairing tumor growth in castration-resistant patient-derived xenograft models [64]. The safety and tolerability of ETC-159 in patients with advanced solid tumors are currently under evaluation in an ongoing phase I clinical trial (NCT02521844). Salinomycin, a polyether ionophore antibiotic, selectively inhibits LRP6 phosphorylation and accelerates its lysosomal degradation, resulting in significant suppression of canonical Wnt signaling and reduction of the PCSC population [143]. Meanwhile, it downregulates key oncogenic drivers, including c-Myc, AR, and ETS transcription factor ERG, induces oxidative stress, compromises cellular antioxidant defenses, and inhibits migration of PCa cells [144]. As key receptors in the canonical Wnt signaling pathway, FZD family members, including FZD2, FZD4, FZD5, and FZD7, are consistently overexpressed in PCa tissues and exert oncogenic effects [145–148]. In contrast, FZD6 is frequently downregulated in PCa and functions as a tumor suppressor; its expression is markedly diminished in PCSCs. Notably, the dietary flavonoid luteolin has been shown to upregulate FZD6 expression, thereby dampening canonical Wnt signaling and attenuating PCSC stemness, and in vivo tumorigenicity [149].

Promoting β-catenin degradation or inhibiting its nuclear translocation and transcriptional activity via the destruction complex remains a key strategy for suppressing canonical Wnt signaling. Phosphorylation of GSK3β at Ser9 inactivates its kinase function, thereby preventing β-catenin phosphorylation and triggering pathway activation. Saikosaponin D and capsaicin have been shown to inhibit canonical Wnt signaling by blocking GSK3β phosphorylation at Ser9, resulting in significant attenuation of PCSC-associated phenotypes. Concurrently, both agents suppress MMP2/9 expression, reverse EMT, and ultimately impair the tumorigenic and invasive potential of CRPC cells [150, 151]. Matairesinol activates GSK3β to promote β-catenin degradation, downregulates c-Myc and CD44 expression, attenuates stem-like properties of CRPC cells, and inhibits EMT by reducing Snail and TWIST protein levels, thereby diminishing metastatic propensity [152]. Sulforaphane, a natural isothiocyanate abundant in cruciferous vegetables, directly binds β-catenin to inhibit canonical Wnt signaling, leading to reduced expression of PCSC markers, decreased PCSC frequency and spheroid-forming capacity, and induction of apoptosis [153]. Ivermectin specifically targets TELO2, thereby suppressing phosphatidylinositol 3-kinase–related kinase (PIKK) activity and reducing cytoplasmic β-catenin accumulation, which ultimately abrogates β-catenin/TCF-mediated transcriptional activation [154]. Eprinomectin, a novel avermectin analog, induces ROS generation and G1/S cell cycle arrest to promote CRPC cell apoptosis while concurrently inhibiting canonical Wnt signaling, reducing the expression of PCSC markers such as ALDH1, SOX2, and NANOG, thus impairing both stemness maintenance and survival of PCSCs [155].

Tankyrase (TNKS), a member of the poly(ADP-ribose) polymerase (PARP) family, is a key positive regulator of canonical Wnt signaling. TNKS1 and TNKS2 catalyze poly(ADP-ribosyl)ation of AXIN, triggering its RNF146-mediated ubiquitination and subsequent proteasomal degradation. This destabilizes the β-catenin destruction complex and potentiates canonical Wnt signaling activity. In PCa, multiple TNKS inhibitors have demonstrated efficacy in suppressing tumor growth and metastasis. For instance, IWR-1 inhibits β-catenin nuclear translocation and concurrently upregulates tight junction proteins, including claudin-5, ZO-1, and ZO-2, thereby restoring barrier integrity and inhibiting PCa cell transendothelial migration and lung metastasis [156]. XAV939 reduces β-catenin accumulation, effectively counteracting canonical Wnt signaling hyperactivation induced by loss of the tumor suppressor MUC15; this suppression impedes EMT and attenuates PCSC properties [157]. Notably, XAV939 enhances antitumor immunity by inhibiting β-catenin nuclear translocation in both PCa cells and CD4⁺ T lymphocytes. Acute exposure accelerates initial tumor cell clearance by lymphocytes, whereas sustained inhibition preserves their cytotoxic function over time [158]. It is worth noting that protein phosphatases, including protein phosphatase 1 (PP1), PP2A, and PP2C, also regulate β-catenin stability [159]. Specifically, the PP2A regulatory subunit PR55α directly binds β-catenin to mediate its dephosphorylation, thereby stabilizing β-catenin and amplifying canonical Wnt signaling [160]; in contrast, the small-molecule inhibitor CS-11 disrupts the PR55α–β-catenin interaction, preventing β-catenin dephosphorylation and reducing levels of active β-catenin. Consequently, CS-11 downregulates c-Myc and Cyclin D1 expression, suppresses CRPC xenograft growth in vivo, and upregulates E-cadherin to reinforce intercellular adhesion, ultimately inhibiting local invasion and distant dissemination [161].

Formation of the β-catenin/TCF transcriptional complex and its recruitment of the histone acetyltransferase CBP constitute the central mechanism through which canonical Wnt signaling activates target gene transcription. As a transcriptional coactivator, CBP is recruited by β-catenin/TCF to target gene promoters, where its intrinsic acetyltransferase activity facilitates chromatin remodeling and transcriptional initiation. Consequently, disrupting this protein–protein interaction suppresses expression of canonical Wnt target genes. CCT036477 is a selective inhibitor of the β-catenin/TCF complex that specifically blocks β-catenin–TCF binding in the nucleus, thereby downregulating oncogenic target genes. Pretreatment of PCa cells with CCT036477 and salinomycin synergistically induces apoptosis by increasing pro-apoptotic/anti-apoptotic protein ratios, and concurrently reduces SOX2 and NANOG expression, significantly decreasing the PCSC population, inhibiting proliferation and migration, and sensitizing cells to cabazitaxel [162]. Similarly, ICG-001 disrupts CBP–β-catenin interaction by competitively binding to CBP while promoting association of β-catenin with p300, thereby reprogramming transcriptional output from stemness and proliferation toward differentiation and apoptosis [163]. ICG-001 has been shown to suppress stem-like marker expression, proliferation, and tumor growth in PCa models via canonical Wnt inhibition; moreover, it restores AR transcriptional activity, thereby enhancing CRPC sensitivity to endocrine therapy [164]. The ALK F1174C activating mutation drives canonical Wnt signaling and cooperates with N-Myc to induce canonical Wnt signaling, converting prostate basal stem cells into aggressive NEPC. ICG-001 treatment impairs NEPC organoid formation, and combined administration with the ALK inhibitor alectinib prevents metastatic dissemination in vivo [165]. PRI-724, another CBP/β-catenin antagonist, selectively inhibits this interaction and has demonstrated efficacy in suppressing EMT, stemness, and metastasis in PCa cells [157]. Collectively, targeting distinct nodes of the canonical Wnt signaling pathway, including ligand secretion, receptor activation, destruction complex integrity, and nuclear transcriptional complexes, represents a promising strategy to deplete PCSCs, reverse EMT, and augment conventional PCa therapies (Table 2).

**Table 2:** Potential canonical Wnt signaling-targeting agents in the treatment of PCSCs

Agents	Category	Targets	Mechanisms and effect	Stage of development	References
ETC-159	Inhibitor	PORCN	Inhibits PORCN to block Wnt palmitoylation, downregulates PCSC markers (AHR, RUNX1) and ROR1, thereby suppressing Wnt5a/ROR1 signaling and tumor growth in castration‑resistant xenografts.	Phase I (NCT02521844)	[64]
Salinomycin		LRP6	Inhibits LRP6 phosphorylation and promotes degradation to block canonical Wnt signaling, thereby decreasing PCSC population and suppressing their colony formation potential.	In vitro	[143]
			Promotes LRP6 degradation to block canonical Wnt signaling, thereby decreasing inhibiting PCSC functions; downregulates c‑Myc/AR/ERG, induces oxidative stress, compromises antioxidant defenses, and inhibits PCa migration.		[144]
Luteolin		FZD6	Upregulates FZD6 to dampen canonical Wnt signaling, reduces PCSC stemness, thereby impairing in vivo tumorigenicity.	In vitro	[149]
Saikosaponin D	Natural compound	GSK3β	Blocks GSK3β Ser9 phosphorylation to suppress canonical Wnt signaling, reduces MMP2/9 expression and PCSC markers, thereby inhibiting PCSC proliferation and metastasis.	In vitro	[150]
Capsaicin			Blocks GSK3β Ser9 phosphorylation to suppress canonical Wnt signaling, reduce PCSC markers, thereby inhibiting PCSC proliferation.		[151]
Matairesinol			Activates GSK3β to promote β‑catenin degradation, downregulates c‑Myc/CD44 and Snail/Twist to suppress stemness and EMT, thereby reducing CRPC metastasis.		[152]
Sulforaphane		β-catenin	Directly binds β‑catenin to inhibit canonical Wnt signaling, reduces PCSC markers, thereby decreasing PCSC frequency and proliferation.	In vitro	[153]
Eprinomectin	Inhibitor		Induces ROS and G1/S arrest to promote apoptosis, suppresses canonical Wnt signaling to reduce PCSC markers (ALDH1, SOX2, NANOG), thereby impairing PCSC stemness and survival.		[155]
CCT036477			Inhibits β‑catenin/TCF binding to reduce PCSC markers (SOX2, NANOG), thereby decreasing PCSC population; synergizes with salinomycin to induce apoptosis, inhibits proliferation/migration, and sensitizes to cabazitaxel.		[162]
Ivermectin		TELO2	Targets TELO2 to inhibit PIKK and cytoplasmic β‑catenin, thereby blocking β‑catenin/TCF transcription.	In vitro/In vivo	[154]
IWR-1		TNKS	Inhibits TNKS to prevent AXIN degradation, decreases β-catenin nuclear translocation, upregulates tight junction proteins (claudin‑5, ZO‑1, ZO‑2) to restore barrier integrity, thereby suppressing PCa transendothelial migration and lung metastasis.	In vitro/In vivo	[156]
XAV939			Reduces β‑catenin to counteract canonical Wnt signaling hyperactivation induced by MUC15 loss, thereby impeding EMT and attenuating PCSC properties.		[157]
			Enhances antitumor immunity by inhibiting β-catenin nuclear translocation in both PCa cells and CD4⁺ T lymphocyte.	In vitro	[158]
CS-11		PP2A	Disrupts PR55α–β‑catenin to block β‑catenin activation, downregulates c‑Myc/Cyclin D1, upregulates E‑cadherin, thereby suppressing CRPC xenograft growth, and inhibiting invasion/metastasis.	In vitro/In vivo	[161]
ICG-001		CBP	Disrupts CBP–β-catenin interaction to inhibit canonical Wnt signaling, thereby suppressing stem‑like marker (ALDH1A1, CD44) expression and tumor growth of PCa cells; restores AR activity to enhance CRPC sensitivity to endocrine therapy.	In vitro/In vivo	[164]
			Impairs NEPC organoid formation and synergizes with alectinib to block metastasis in vivo.		[165]
PRI-724			Disrupts CBP–β-catenin interaction to inhibit canonical Wnt signaling, thereby suppressing EMT, stemness, and metastasis in PCa cells.		[157]

#### Challenges and opportunities of targeting Wnt signaling for PCSC inhibition

Although targeting the canonical Wnt signaling holds promise for suppressing PCSCs, its clinical translation faces multiple significant challenges, with systemic toxicity representing a primary limiting factor. Under physiological conditions, canonical Wnt signaling is indispensable for maintaining normal stem cell self-renewal, tissue regeneration, and organ homeostasis; excessive inhibition therefore broadly compromises vital organ functions, particularly in the gastrointestinal tract, bone, and hematopoietic systems. For instance, conditional deletion of *CTNNB1* in intestinal epithelial cells of adult mice rapidly suppresses crypt proliferation and downregulates canonical Wnt target gene expression, resulting in progressive loss of intestinal stem and undifferentiated progenitor cells, structural degeneration of crypts, and impaired epithelial turnover [166]. This mechanism directly accounts for the high incidence of gastrointestinal adverse events observed with Wnt inhibitors. Canonical Wnt signaling promotes osteoblast differentiation from mesenchymal progenitors via LRP5/6 co-receptors, enhances osteoblast proliferation, survival, and bone matrix synthesis, and upregulates osteoprotegerin to indirectly suppress osteoclastogenesis, thereby maintaining bone homeostasis. Functional impairment of this axis leads to deficient bone formation, markedly reduced bone density and mechanical strength, and clinically manifests as osteoporosis and increased fracture risk [167]. In hematopoiesis, canonical Wnt signaling regulates stem and progenitor cell output in a dose-dependent manner. Genetic ablation of Wnt3a depletes fetal liver hematopoietic stem and progenitor cells, causing embryonic lethality by embryonic day 12.5 [168], and LEF1-deficient mice display impaired pro-B cell expansion and survival [169]. Clinical evidence further underscores the safety concerns associated with systemic Wnt pathway inhibition, particularly when targeting upstream regulators such as PORCN or Wnt ligand secretion. A Phase 1 trial (NCT01351103) of the PORCN inhibitor LGK974 in advanced solid tumors demonstrated limited antitumor activity, with only 16% of patients achieving stable disease; the most frequent adverse event was dysgeusia (50% of patients), which was strongly associated with reduced AXIN2 suppression [170]. Similarly, a Phase Ib/II study (NCT02278133) evaluating LGK974 combined with encorafenib and cetuximab in BRAF V600E-mutant metastatic colorectal cancer reported an overall response rate of 10%, yet 75% of patients experienced serious adverse events, including bone fractures, hypercalcemia, and pleural effusion, with 45% developing bone toxicities that led to study discontinuation [171]. In contrast, ipafricept (a FZD8-Fc fusion protein) exhibited favorable tolerability across solid tumor types (NCT01608867); however, dose-limiting fragility fractures occurred at higher exposures, and approximately 20% of patients developed dysgeusia, decreased appetite, fatigue, and muscle spasms. At the recommended phase 2 dose, durable disease stabilization (> 6 months) was observed in subsets of patients with desmoid tumors and germ cell tumors [172]. However, a dose-escalation Phase I study (NCT04505839) of the tankyrase inhibitor basroparib in advanced colorectal cancer confirmed a favorable safety profile: despite a disease stabilization rate of 23.5%, no dose-limiting toxicities or treatment-related deaths occurred, and mild/moderate fatigue and nausea were the common adverse events [173]. This differential safety profile likely reflects mechanistical distinctions. LGK974 inhibits PORCN to block secretion of all Wnt ligands, thereby disrupting both canonical and non-canonical Wnt signaling, whereas ipafricept sequesters extracellular Wnt ligands selectively via FZD8, and basroparib inhibits tankyrase-mediated AXIN1/2 degradation without abolishing physiological Wnt signaling, resulting in more constrained pathway modulation and an improved therapeutic efficacy. Therefore, Wnt signaling-targeted therapy must be guided by molecular stratification: agents should be selected based on the specific Wnt-altering lesion driving tumorigenesis (e.g., R-spondin fusions, *RNF43* mutations, *APC* loss) rather than applied uniformly across all Wnt-activated tumors. Notably, advances in single‑cell spatial multi‑omics technologies now enable high‑resolution, cell‑type–resolved mapping of Wnt signaling activity within the TME, which facilitates the identification of context‑specific Wnt signaling alterations across malignant, stromal, and immune subpopulations and thus informs the rational design of precision‑targeted interventions.

Second, signaling pathway redundancy fundamentally limits the efficacy of monotherapy targeting the canonical Wnt signaling in suppressing PCSCs. In PCa, oncogenic drivers beyond AR, including PTEN loss–induced PI3K/AKT/mTOR hyperactivation and IL‑6–stimulated STAT3 signaling, can sustain PCSC stemness independently of canonical Wnt signaling [174, 175]. This redundancy operates through two complementary mechanisms. First, convergent regulation of shared downstream effectors, such as STAT3- or AKT-mediated c‑Myc overexpression, which transcriptionally activates stemness-related genes including ALDH1 and SOX2 [46, 176]; and second, c‑Myc–independent functional compensation, whereby STAT3 and AKT directly regulate PCSC self‑renewal, EMT, and metastatic competence even when canonical Wnt signaling is suppressed [116]. This represents a fundamental constraint in precision oncology. Consequently, therapeutic strategies are shifting toward rational combinations that co‑target parallel stemness‑maintaining pathways, either alone or synergistically with standard‑of‑care modalities such as ADT or chemotherapy. Preclinically, the STAT3 inhibitor napabucasin (BBI‑608) robustly impairs PCSC self‑renewal capacity [177]. Its integration into micellar nanodrugs, co‑formulated with indocyanine green and Acupa ligand, enables tumor‑selective photothermal ablation of both bulk PCa cells and PCSCs while preventing recurrence [178]. Clinically, napabucasin combined with gemcitabine and nab‑paclitaxel achieved a 93% disease control rate in metastatic pancreatic cancer, supporting advancement to Phase 3 (NCT02993731) [179]. Likewise, the AKT inhibitor ipatasertib plus abiraterone significantly prolonged progression‑free survival in PTEN‑deficient mCRPC (NCT03072238); although PCSC markers were not prospectively assessed, the essential role of PI3K/AKT signaling in PCSC maintenance strongly supports its functional impact on this compartment [180]. Furthermore, the BET inhibitor ZEN‑3694 suppresses c‑Myc transcription via BRD4 inhibition. A Phase Ib/IIa trial (NCT02711956) combining ZEN‑3694 with enzalutamide demonstrated acceptable tolerability and preliminary antitumor activity in mCRPC, and preclinical validation confirms that ZEN‑3694 caused PCSC exhaustion and diminishes tumorigenic capacity in vivo [181, 182]. Collectively, these data indicate that overcoming redundancy–driven stemness maintenance necessitates combinatorial targeting, not merely additive inhibition but rationally designed regimens that simultaneously disrupt multiple convergent and parallel signaling nodes sustaining PCSC activity.

Additionally, the context‑dependent functional duality of specific Wnt pathway components presents a distinct therapeutic challenge. GSK3β exemplifies this complexity: it acts as a tumor suppressor within the destruction complex of canonical Wnt signaling by phosphorylating β‑catenin for proteasomal degradation, and it also directly binds and phosphorylates the AR, thereby attenuating AR transcriptional activity and restraining PCa progression [183]. Conversely, in PTEN‑null contexts, AKT‑mediated inhibitory phosphorylation of GSK3β abrogates its kinase activity, leading to β‑catenin stabilization and nuclear accumulation, which promotes PCa progression [184]. Moreover, GSK3β exerts β‑catenin–independent anti‑apoptotic effects. Tumor necrosis factor-related apoptosis-inducing ligand (TRAIL) stimulation induces activating Tyr216 phosphorylation of GSK3β, which then suppresses TRAIL‑induced apoptosis in PCa cells, while pharmacologic GSK3β inhibition conversely restores sensitivity to TRAIL‑ and CDDO‑Me–induced apoptosis [185, 186]. Thus, indiscriminate GSK3β inhibition may antagonize the pro‑apoptotic efficacy of Wnt signaling‑targeted therapy. Therefore, combining Wnt pathway inhibitors with GSK3β inhibitors simultaneously suppresses pro‑proliferative signaling and reverses apoptosis inhibition, synergistically enhancing therapeutic efficacy in targeted interventions. Similarly, the non‑canonical ligand Wnt5a displays receptor‑contextual duality. In ROR2‑expressing PCa cells, Wnt5a exerts tumor‑suppressive effects by activating Hippo signaling, whereas in ROR2‑null cells it promotes cancer progression through inhibition of this pathway [101]. Accordingly, ROR2 expression level serves as a predictive biomarker for Wnt5a‑directed therapies. Collectively, these findings indicate that indiscriminate Wnt signaling inhibition may inadvertently compromise endogenous tumor‑suppressive Wnt signaling.

Achieving precise elimination of PCSCs through targeted modulation of Wnt signaling demands a coordinated, multi‑dimensional strategy. First, tumor‑specific genetic vulnerabilities can be exploited for synergistic blockade; for example, in PTEN‑deficient tumors, combined AKT and canonical Wnt signaling inhibition disrupts the co‑dependence of PCSCs on PI3K and Wnt signaling. Second, integrated molecular phenotyping enables reliable discrimination of PCSCs from normal prostate basal stem cells. The latter are characterized by the expression of CK5/14, p63, CD44, α2β1 integrin, CD133, and Trop2, whereas PCSCs frequently retain CD44, α2β1 integrin, and CD133 but typically lack p63 and aberrantly overexpress stemness‑associated markers such as TRA‑1‑60 and CD166 [187, 188]. CD44v6⁺ cells also exhibit PCSC properties, further refining target cell identification. Third, PCa cell‑specific delivery systems should be developed to minimize off‑target toxicity. PSMA‑targeted ligand‑drug conjugates, for instance, efficiently enrich β‑catenin/TCF transcriptional inhibitors such as ICG‑001 and PRI‑724 in PCa cells, particularly in the castration‑resistant setting. Hyaluronic acid‑modified or anti‑CD44v6 antibody‑conjugated nanocarriers enable active PCSC targeting and co‑delivery of STAT3 inhibitors (e.g., napabucasin) or Wnt signaling inhibitors, with photothermal‑triggered release further enhancing precision killing. micellar nanodrug studies have confirmed the potential of this strategy to eradicate CSCs and prevent recurrence [178]. Moreover, prodrugs of Wnt signaling inhibitors designed to be cleaved by prostate‑specific proteases such as PSA restrict active drug release to the prostate TME. These delivery approaches selectively eliminate PCSCs while preserving physiological Wnt signaling in normal tissues, offering a path toward safe and complete tumor eradication. Although most canonical Wnt pathway inhibitors remain in preclinical development, the identification of naturally derived bioactive compounds and drug repurposing efforts continue to expand the therapeutic pipeline for PCSC-directed intervention. Concurrently, advances in single‑cell spatiotemporal omics, next‑generation precision delivery platforms, and refined drug safety assessment frameworks are strengthening the translational foundation for precision PCSC-targeted therapy.

## Conclusions

In summary, aberrant activation of canonical Wnt signaling serves as a central driver of PCSC self-renewal, lineage plasticity, EMT, and bone metastasis. Natural products and small‑molecule inhibitors targeting distinct nodes of this pathway have demonstrated substantial preclinical efficacy in suppressing stemness, reversing therapeutic resistance, and inhibiting metastasis, and several have advanced to clinical trials. Nevertheless, their clinical application remains constrained by systemic toxicity, pathway redundancy, and insufficient target specificity. As understanding of PCSC regulatory mechanisms deepens and medicinal chemistry and delivery technologies advance, targeting canonical Wnt signaling is progressively overcoming the therapeutic impasse in CRPC. Future strategies will leverage patient‑derived organoids, single‑cell sequencing, and liquid biopsy to monitor dynamic Wnt signaling activity in PCSCs in real time, enabling identification of CRPC subtypes most likely to respond. Rationally combining highly selective Wnt signaling inhibitors with ADT, epigenetic modulators, and immune checkpoint inhibitors holds strong promise for improving efficacy and overcoming resistance. Building on this, prostate tissue‑targeted precision delivery systems that concentrate therapeutics within the PCSC niche while sparing systemic exposure offer a viable route toward personalized, curative treatment for CRPC and significant improvement in patient outcomes.

## Data Availability

Not applicable.

## Abbreviations

ABC: ATP binding cassette
ABCG2: ATP binding cassette subfamily G member 2
ADT: Androgen deprivation therapy
AHR: Aryl hydrocarbon receptor
ALDH: Aldehyde dehydrogenase
ALDH1A1: Aldehyde dehydrogenase 1 family member A1
AMPK: AMP activated protein kinase
APC: Adenomatous polyposis coli
AR: Androgen receptor
AR-V7: Androgen receptor splice variant 7
AXIN: Axis inhibition protein
BCL9: B-cell CLL/lymphoma 9 protein
BMAL1: Basic helix-loop-helix ARNT like 1
β-TrCP: β-transducin repeat-containing protein
CAFs: Cancer associated fibroblasts
CAMK1D: Ca²⁺/calmodulin-dependent protein kinase 1D
CBP: CREB-binding protein
CXCL12: C-X-C motif chemokine ligand 12
CCL5: C-C motif chemokine ligand 5
CCR5: C-C motif chemokine receptor 5
CD44v6: CD44 variant 6
circRNAs: Circular RNAs
CK19: Cytokeratin 19
CK1α: Casein kinase 1α
CKS2: Cyclin-dependent kinase regulatory subunit 2
CRPC: Castration-resistant prostate cancer
CRY: Cryptochrome circadian regulator
CSCs: Cancer stem cells
CST1: Cystatin-1
CTCs: Circulating tumor cells
ctDNA: Circulating tumor DNA
CXCR4: C-X-C motif chemokine receptor 4
DAB2IP: Disabled homolog 2-interacting protein
DDX3X: DEAD-box helicase 3 X-linked
DHT: Dihydrotestosterone
DKK: Dickkopf
DVL: Dishevelled
ECM: Extracellular matrix
EGR1: Early growth response 1
EMT: Epithelial–mesenchymal transition
EZH2: Enhancer of zeste homolog 2
FBXW2: F-box and WD repeat domain containing 2
FASN: Fatty acid synthase
FOXO3a: Forkhead box O3
FZD: Frizzled receptor
GLS: Glutaminase
GSK3β: Glycogen synthase kinase 3β
HDAC2: Histone deacetylase 2
IGF1: Insulin-like growth factor 1
ITCH: Itchy E3 ubiquitin protein ligase
KLF4: Kruppel-like factor 4
KLK3: Kallikrein related peptidase 3
KMT2B: Lysine methyltransferase 2B
LATS1/2: Large tumor suppressor kinase 1
LEF: Lymphoid enhancer factor
lncRNAs: Long non-coding RNAs
LRP5/6: Low-density lipoprotein receptor-related protein 5/6
mCRPC: Metastatic castration-resistant prostate cancer
MDR1: Multidrug resistance protein 1
MDSCs: Myeloid-derived suppressor cells
MICs: Metastasis-initiating cells
MMP2: Matrix metallopeptidase 2
MSCs: Mesenchymal stem cells
MSS: Microsatellite-stable
MST1/2: Macrophage stimulating 1/2
NAMPT: Nicotinamide phosphoribosyltransferase
ncRNA: Non-coding RNA
NEPC: Neuroendocrine prostate cancer
NFIL3: Nuclear factor interleukin-3-regulated protein
OCT4: Octamer-binding transcription factor 4
ODAM: Odontogenic ameloblast-associated protein
OPG: Osteoprotegerin
OS: Overall survival
Osx: Osterix
PARP: Poly(ADP-ribose) polymerase
PCa: Prostate cancer
PCSCs: Prostate cancer stem cells
PER: Period circadian regulator
PIKK: Phosphatidylinositol 3-kinase–related kinase
PSMA: Prostate-specific membrane antigen
PORCN: Porcupine
PP1: Protein phosphatase 1
PP2A: Protein phosphatase 2 A
PRC2: Polycomb repressive complex 2
PROX1: Prospero homeobox protein 1
RANK: Receptor activator of nuclear factor-κB
RANKL: RANK ligand
RBM3: RNA-binding motif protein 3
RNF43: Ring finger protein 43
RNS: Reactive nitrogen species
ROR1: Receptor tyrosine kinase-like orphan receptor 1
ROS: Reactive oxygen species
RSPOs: R-spondins
RTK: Receptor tyrosine kinase
RUNX1: Runt-related transcription factor 1
SFRP1: Secreted frizzled-related protein 1
SIX2: SIX homeobox 2
SOX2: SRY-box transcription factor 2
TAMs: Tumor-associated macrophages
TGF-β: Transforming growth factorβ
3′-UTRs: 3′-untranslated regions
TLE: Groucho/transducin-like enhancer of split
TME: Tumor microenvironment
TMPRSS2: Transmembrane serine protease 2
TNKS: Tankyrase
TRAIL: Tumor necrosis factor-related apoptosis-inducing ligand
TWIST1: Twist related protein 1
ZEB1: Zinc finger E-box binding homeobox 1
ZNRF3: Zinc and ring finger 3
